# Influenza Virus Infects Epithelial Stem/Progenitor Cells of the Distal Lung: Impact on Fgfr2b-Driven Epithelial Repair

**DOI:** 10.1371/journal.ppat.1005544

**Published:** 2016-06-20

**Authors:** Jennifer Quantius, Carole Schmoldt, Ana I. Vazquez-Armendariz, Christin Becker, Elie El Agha, Jochen Wilhelm, Rory E. Morty, István Vadász, Konstantin Mayer, Stefan Gattenloehner, Ludger Fink, Mikhail Matrosovich, Xiaokun Li, Werner Seeger, Juergen Lohmeyer, Saverio Bellusci, Susanne Herold

**Affiliations:** 1 Department of Internal Medicine II, Universities Giessen & Marburg Lung Center (UGMLC), member of the German Center for Lung Research (DZL), Giessen, Germany; 2 Excellence Cluster Cardio-Pulmonary System (ECCPS), Universities Giessen & Marburg Lung Center (UGMLC), member of the German Center for Lung Research (DZL), Giessen, Germany; 3 Department of Pathology, Justus-Liebig-University Giessen, Giessen, Germany; 4 Institute of Pathology and Cytology, Wetzlar, Germany, member of the German Center for Lung Research (DZL), Giessen, Germany; 5 Institute of Virology, Philipps University, Marburg, Germany; 6 College of Pharmacy, Wenzhou Medical University, Wenzhou, Zhejiang, China; 7 Department of Lung Development and Remodelling, Max Planck Institute for Heart and Lung Research, Bad Nauheim, Germany; 8 College of life and Environmental sciences and College of Chemistry and Materials Engineering, Wenzhou University, Wenzhou University town, Zhejiang, China; St. Jude Children's Research Hospital, UNITED STATES

## Abstract

Influenza Virus (IV) pneumonia is associated with severe damage of the lung epithelium and respiratory failure. Apart from efficient host defense, structural repair of the injured epithelium is crucial for survival of severe pneumonia. The molecular mechanisms underlying stem/progenitor cell mediated regenerative responses are not well characterized. In particular, the impact of IV infection on lung stem cells and their regenerative responses remains elusive. Our study demonstrates that a highly pathogenic IV infects various cell populations in the murine lung, but displays a strong tropism to an epithelial cell subset with high proliferative capacity, defined by the signature EpCam^high^CD24^low^integrin(α6)^high^. This cell fraction expressed the stem cell antigen-1, highly enriched lung stem/progenitor cells previously characterized by the signature integrin(β4)^+^CD200^+^, and upregulated the p63/krt5 regeneration program after IV-induced injury. Using 3-dimensional organoid cultures derived from these epithelial stem/progenitor cells (EpiSPC), and *in vivo* infection models including transgenic mice, we reveal that their expansion, barrier renewal and outcome after IV-induced injury critically depended on Fgfr2b signaling. Importantly, IV infected EpiSPC exhibited severely impaired renewal capacity due to IV-induced blockade of β-catenin-dependent Fgfr2b signaling, evidenced by loss of alveolar tissue repair capacity after intrapulmonary EpiSPC transplantation *in vivo*. Intratracheal application of exogenous Fgf10, however, resulted in increased engagement of non-infected EpiSPC for tissue regeneration, demonstrated by improved proliferative potential, restoration of alveolar barrier function and increased survival following IV pneumonia. Together, these data suggest that tropism of IV to distal lung stem cell niches represents an important factor of pathogenicity and highlight impaired Fgfr2b signaling as underlying mechanism. Furthermore, increase of alveolar Fgf10 levels may represent a putative therapy to overcome regeneration failure after IV-induced lung injury.

## Author Summary

Influenza Virus (IV) pneumonia causes disruption of the alveolar epithelial barrier, leading to edema formation which severely affects gas exchange. Recovery after IV-induced acute lung injury includes not only innate immune responses to clear virus infection, but also tissue renewal processes to restore the alveolar barrier. We demonstrate that highly pathogenic IV particularly infect cells of the epithelial stem/progenitor cell niche, which significantly impairs repair processes within the damaged lung, as demonstrated by transplantation experiments using intrapulmonary delivery of infected versus non-infected stem cells into IV-injured mice. Analyses of the underlying virus-host interactions reveal that IV infection of epithelial stem/progenitor cells results in blockade of a pathway involving Wnt/β-catenin and fibroblast growth factor receptor 2b (Fgfr2b) activation within a regenerative epithelial-mesenchymal cell signaling network. Our data therefore suggest that tropism of IV to the distal lung stem cell niche represents an important factor of pathogenicity. Therapeutic intrapulmonary application of excess Fgf10 to induce Fgfr2b signaling in the non-infected stem cell niche was found to counteract IV-induced repair failure and to restore barrier function, representing a promising treatment to foster epithelial cell regeneration after IV-induced injury.

## Introduction

Influenza viruses (IV) may cause primary viral pneumonia in humans with rapid progression to lung failure and fatal outcome, and treatment options for this sometimes devastating disease are limited [1, 2]. Histopathology and clinical features of IV-induced lung injury in humans resemble those of other forms of ARDS (acute respiratory distress syndrome) and are characterized by apoptotic and necrotic airway and alveolar epithelial cell death, loss of pulmonary barrier function and severe hypoxemia [1, 3, 4]. IV primarily infect cell subsets of the upper and lower respiratory tract. In the latter, these are particularly ciliated and goblet cells, club cells and alveolar epithelial cells type II (AECII) [5–7]. Injury of lung epithelial cells is induced by both direct viral cytopathogenicity and unbalanced immune responses [8–11]. The initiation of well-coordinated programs of inflammation termination and of regeneration of the injured distal lung epithelium are a prerequisite for the re-establishment of proper gas exchange. Absence or imbalance of these responses may at best result in chronically organizing infiltrates and aberrant or excess remodeling with tissue fibrosis, associated with long-term pulmonary organ dysfunction in ARDS survivors [12, 13], or in fatal outcome at worst. However, the cellular communication patterns and molecular networks underlying regeneration of the distal lung compartment after severe pathogen-associated injury are incompletely understood to date. In particular, the distinct mechanisms of interaction between injury-causing pathogens with components of regenerative signaling pathways within the lung stem cell niche, determining outcome of the repair response, have not been studied in detail.

Alveolar re-epithelialization after injury was shown to involve different populations of endogenous, organ-resident stem/progenitor cells, which express lineage markers of distal lung epithelium such as club cell-specific protein (CC10/scgb1a1) or surfactant protein C (SPC/sftpc), are quiescent under normal conditions and proliferate during repair [14]. More recent reports revealed that intrinsically committed distal airway stem cells (DASC) expressing keratin 5 (krt5) and the transcription factor p63 were found to contribute to *de novo* generation of both bronchiolar and alveolar tissue after formation of cell “pods” in a murine model of IV infection [15, 16]. Vaughan et al. defined lineage-negative, integrin(β4)^+^CD200^+^ epithelial progenitors as the source of p63/krt5^+^ amplifying cells regenerating airways and alveoli, highlighting integrin(β4)^+^CD200^+^ epithelial cells as important progenitors regenerating the distal lung following IV-induced injury [17].

During regeneration processes, the lung stroma likely plays a key role by maintaining the distinct microenvironment of the stem cell niche, involving extracellular matrix, direct cell-cell contacts and autocrine or paracrine mediators. These signals initiate and co-ordinate self-renewal, fate determination and terminal differentiation of stem/progenitor cells. Different subsets of resident lung stromal/mesenchymal cells have been attributed a role in these processes, including parabronchial smooth muscle cells [18], Sca-1^high^ lung mesenchymal cells [19, 20] or a human vimentin^+^ lung fibroblast population [21]. Signals involved in these cross-talk events include, among others, the paracrine fibroblast growth factors (Fgfs), which regulate cell survival, proliferation, differentiation, and motility. In particular, Fgf7 and Fgf10 and their common tyrosine kinase receptor Fgfr2b (fibroblast growth factor receptor 2b), are indispensable for distal lung development including branching morphogenesis [19, 22–24]. Fgfr2b signaling is also re-activated in stem cell niches of the adult lung after different forms of injury to regenerate the epithelium [23, 25, 26]. The regulation of ligand and receptor expression of the Fgf7/10-Fgfr2b network in the context of lung repair after infectious injury, however, is not well understood.

In the current study, we demonstrate that a highly proliferating EpCam^high^CD24^low^integrin(α6β4)^high^CD200^+^ distal lung epithelial cell population represents a primary target of pathogenic IV. This population highly enriched cells expressing key characteristics of distal lung epithelial stem/progenitor cells mediating bronchiolar and alveolar repair. Of note, IV tropism to these cells significantly reduced their regeneration capacity by impairment of β-catenin-dependent Fgfr2b signaling. These data for the first time demonstrate that the extent of lung stem/progenitor cell infection by IV is a hallmark of pathogenicity as it critically impacts on lung regeneration capacity after severe IV injury. Moreover, IV-induced regeneration failure could be counteracted by intratracheal application of excess recombinant Fgf10, suggesting recruitment of the non-infected Fgfr2b^high^ stem cell fraction for repair as putative novel treatment strategy to drive organ regeneration in patients with IV-induced ARDS.

## Results

### Influenza viruses target epithelial cell subsets of the distal murine lung to different extent after intratracheal infection

**Fig 1:**
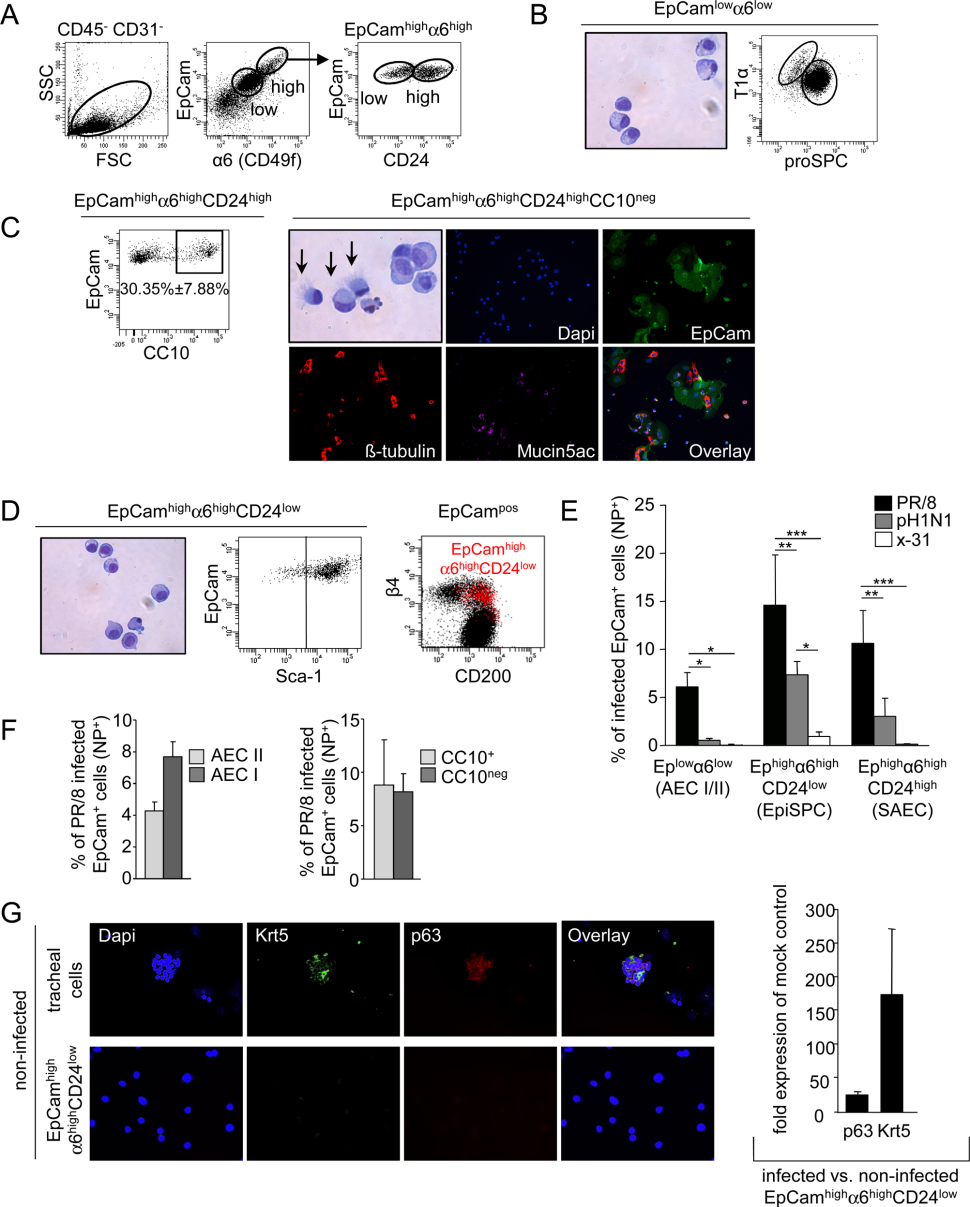
Characterization of distal lung epithelial cell subpopulations and analysis of their infection rates *in vivo*. (A) Gating strategy of three epithelial cell subsets in CD31 and CD45 depleted lung homogenates of wt mice according to the expression of EpCam, α6 integrin and CD24. (B) Pappenheim stained cytospins of flow-sorted EpCam^low^α6^low^ epithelial cells and flow cytometric subgating of this fraction with proSPC and T1α. (C) Characterization of the EpCam^high^α6^high^CD24^high^ subpopulation by flow cytometry reveals a CC10^+^ and a CC10^neg^ fraction. Pappenheim stained cytospins of flow-sorted EpCam^high^α6^high^CD24^high^CC10^neg^ epithelial cells (arrows indicate ciliated cells) and immunofluorescence stainings of this cell subset with mucin5ac and β-tubulin after 4d of culture. (D) Flow-sorted and Pappenheim stained cytospins of the EpCam^high^α6^high^CD24^low^ epithelial cell population (left). Further flow cytometric phenotype characterization of the EpCam^high^α6^high^CD24^low^ population revealed that it is Sca-1^+^ (middle) and localizes to the β4 integrin^+^ and CD200^+^ fraction of EpCam^+^ cells (right, EpCam^high^α6^high^CD24^low^ population depicted in red). (E-F) Wt mice were infected with 500pfu of the indicated influenza virus strains and the fractions of influenza virus infected (nucleoprotein^+^, NP^+^) cells of the different EpCam^+^ subpopulations were determined by FACS at d4 pi. (G) Cytospins of the flow-sorted EpCam^high^α6^high^CD24^low^ population or of tracheal digests (positive control) from uninfected mice were stained for krt5 and p63 (left). Quantification of p63 and krt5 mRNA levels of flow-sorted EpCam^high^α6^high^CD24^low^ at d14 pi from PR/8 infected mice (right). Bar graphs represent fold induction compared to mock-infected controls. Bar graphs represent means ± SD of n = 4 independent experiments; * *p*<0.05; **p<0.01; ***p<0.001.

It is well established that IV infect different subsets of the airways and alveoli, particularly ciliated and goblet cells, club cells and AECII [5–7]. However, recent advances in the field resulted in the definition of more specialized subsets of lung epithelial cells, some of which display stem/progenitor cell characteristics and contribute to repair of the injured organ [17, 19, 27]. To address which of these epithelial cell compartments were infected by IV, we fractionated distal lung epithelial cells into different subsets, after dissection of large airways and vessels and depletion of leukocytes and endothelial cells, according to surface expression levels of EpCam and integrin α6 [19], and the lineage markers CD24 (differentiated airway epithelial cells) [19], CC10 (club cells), pro-SPC (AEC II) and T1α (AEC I), by flow cytometry. We identified a high-frequent EpCam^low^α6^low^ fraction (91.3 ± 1.8%) and a low frequent EpCam^high^α6^high^ fraction, the latter of which consisted of a CD24^low^ and CD24^high^ population (1.7 ± 0.3% and 6.3 ± 1.8%, respectively, Fig 1A). The majority of the most abundant EpCam^low^α6^low^ cells showed a granular cytoplasm typically observed in AEC II, with approximately 95% of the cells expressing the AEC II signature pro-SPC^high^T1α^neg^ and around 5% expressing an AEC I signature (SPC^neg^T1α^+^) (Fig 1B). EpCam^high^α6^high^CD24^high^ cells contained pro-SPC^neg^CC10^neg^ differentiated small airway epithelial cells (SAEC, 70%), composed of both β-tubulin^+^ ciliated and mucin5AC^+^ goblet cells, and pro-SPC^neg^CC10^+^ club cells (30%) (Fig 1C). EpCam^high^α6^high^CD24^low^ cells were cells of homogeneous morphology and stained positive for the stem cell antigen Sca-1^+^ (Fig 1D).

To analyse which of these epithelial cell subset were targeted by IV, we infected C57BL/6 mice using 500pfu of IV strains of increasing pathogenicity, i.e. low-pathogenic H3N2 (x-31), pandemic pH1N1 strain (A/Hamburg/04/09), causing mild to moderate lung injury at this dose in mice, and the highly pathogenic mouse-adapted PR/8 strain [28, 29]. Quantification of the infection rates by staining for IV nucleoprotein (NP) revealed that EpCam^low^α6^high^CD24^high^ and EpCam^low^α6^low^ cells were infected with a frequency of ∼11% and ∼6%, respectively, by d4 pi after PR/8 infection, a time point where PR/8 replication in the lung reaches a peak [28]. Subfractionation into differentiated alveolar and airway epithelial cells revealed that rates of PR/8 infection in AEC I and AEC II ranged at ∼8% and ∼4%, whereas club cells and ciliated/goblet cells displayed similar PR/8 infection rates of ∼10%. Of note, EpCam^high^α6^high^CD24^low^ cells were infected by IV to high amounts (around 15% of all EpCam^high^α6^high^CD24^low^ after PR/8 infection), and the proportion of infected epithelial cell subsets at d4 pi correlated with the level of pathogenicity of the IV strain used (Fig 1E and 1F). To further address whether PR/8 revealed increased tropism to EpCam^high^α6^high^CD24^low^ cells, AEC, SAEC and EpCam^high^α6^high^CD24^low^ cells were flow-sorted, seeded into culture plates at equal numbers and infected *ex vivo* with x-31, PR/8 and pH1N1 at an MOI of 2, respectively. After one replication cycle (6h), excluding de novo infection by progeny virions, the infection rate was determined, reflecting the capacity of each virus strain to infect the respective cell population. Similar to our *in vivo* results, we observed that PR/8 reveals an enhanced tropism to EpCam^high^α6^high^CD24^low^ cells (S1 Fig).

### Highly infectable EpCam^high^α6^high^CD24^low^Sca-1^pos^ cells reveal epithelial stem cell characteristics and generate lung-like organoids in an Fgf10-dependent manner

Given that the EpCam^high^α6^high^CD24^low^Sca-1^pos^ cells which revealed the highest rates of infection were previously described as epithelial stem/progenitor population giving rise to airway and alveolar epithelium [19], we aimed to further characterize their phenotype and stemness properties. Further analyses using established stem cell markers [17] revealed that they were integrin β4^+^CD200^+^, a signature which has been confined to a distal lung stem cell phenotype known to engage the krt5/p63 regeneration program [17] (Fig 1D). In accordance, these cells were negative for krt5 and p63 in healthy lungs, but highly upregulated krt5 and p63 gene expression after IV-induced injury (Fig 1G), suggesting that they contain epithelial stem/progenitor cells (EpiSPC).

To verify these stem/progenitor cell characteristics *ex vivo*, EpCam^+^ cell fractions were flow-sorted and seeded in organotypic 3D cultures [30]. As opposed to AEC I/II, and SAEC/club cells, EpiSPC developed typical large organoid spheres with cystic or saccular outgrowth in the presence of the growth factors Fgf10 and Hgf (hepatocyte growth factor), a characteristic feature of lung stem/progenitor cells [19] (S2A Fig). A robust clonogenic potential of EpiSPC was demonstrated by repetitive cycles of serial passaging of digested organospheres by single-cell sorting and detection of *de novo* sphere formation after one week, respectively (S2B Fig), and by use of clonality assays where tdtomato^+^ EpiSPC were mixed with wildtype (wt) EpiSPC at a defined ratio and cultured in matrix, resulting in pure tdtomato^+^ and tdtomato^neg^ colonies indicative of clonal expansion (S2C Fig).

Given that Fgf10-Fgfr2b signaling is indispensable for epithelial stem cell outgrowth *ex vivo* [18, 23, 30], we aimed to further define whether EpiSPC expansion and differentiation were Fgf10-dependent. To understand cellular cross-talk mechanisms involved in activation of the regenerative Fgfr2b axis after IV-induced lung injury we sought to define the predominant cellular source of Fgfr2b ligands, Fgf7 and 10 under homeostatic conditions and in the acute phase of IV-induced lung injury, at the peak of EpiSPC proliferation (d7 pi). Lung digests of mock- or PR/8-infected mice (d7 pi) were therefore fractionated by FACS sorting into four main lineages, including endothelial cells (CD31^+^CD45^neg^EpCam^neg^, R1), leukocytes (CD31^neg^CD45^+^EpCam^neg^, R2), epithelial cells (CD31^neg^CD45^neg^EpCam^+^, R3) and CD31^neg^CD45^neg^EpCam^neg^Sca-1^high^ cells (S3A Fig, left, R4). mRNA expression of Fgfr2b ligands in these four populations revealed that both Fgf7 and Fgf10 expression was significantly increased in the R4 fraction (CD31^neg^CD45^neg^EpCam^neg^Sca-1^high^) compared to the other three major lineages of the lung (endothelial cells, epithelial cells, leukocytes), independent of IV infection (S3A Fig, right). Previous data suggested that Sca-1^high^ expression in EpCam^neg^ cells was associated with the fibroblast lineage [20]. To address whether Fgf10-expressing resident mesenchymal cells (rMCs) would support organosphere generation, flow-sorted EpiSPC and rMC were co-cultured for several days in absence of growth factor supplementation. As shown in S3B Fig, presence of rMC was sufficient to drive early organosphere formation at d5 of culture. Furthermore, rMCs mediated saccular outgrowth of EpiSPC spheres at d10 and formation of lung-like structures at d16 of culture, as compared to EpiSPC mono-cultures in supplemented medium, and this response was abrogated early in the cystic phase when Fgf10 was blocked by neutralizing antibodies (S3D Fig). Of note, co-culture of EpiSPC with either flow-sorted CD45^+^ (R2) or CD31^+^ (R1) cells did not result in organosphere formation (S3C Fig). Finally, lung-like structures derived from EpiSPC-rMA co-cultures significantly upregulated markers of terminal airway and alveolar cell differentiation, such as T1α and β-tubulin (S3E Fig). Together, these data indicate that EpiSPC both self-renew and differentiate to distal lung epithelial cell subsets in an Fgf10-dependent manner during organotypic culture.

### High renewal capacity of EpiSPC after IV-induced lung injury is mediated by the Fgf10-Fgfr2b axis

**Fig 2:**
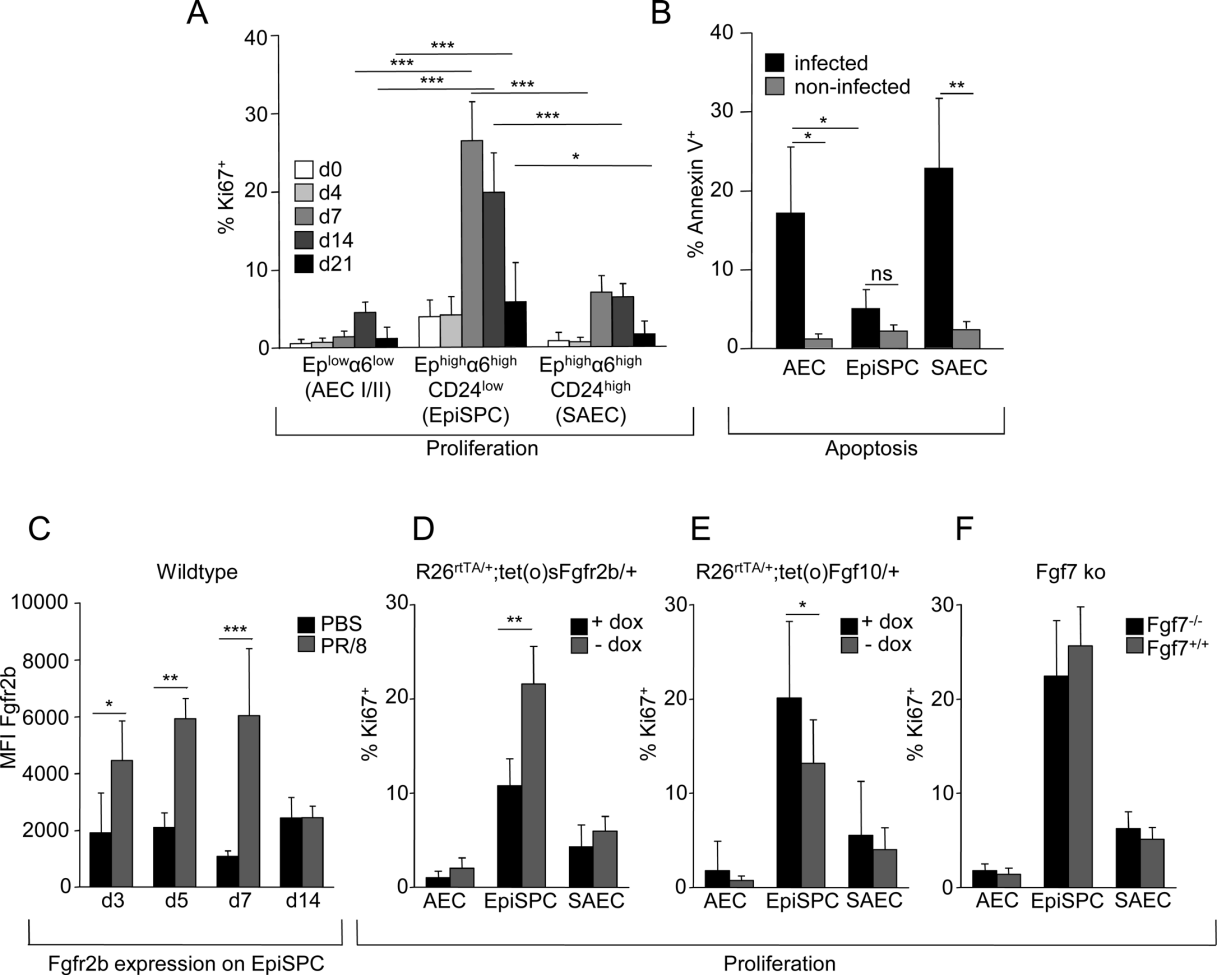
EpiSPC are resistant to apoptosis and show a high proliferative response after PR/8 infection which is mediated by Fgf10/Fgfr2b signaling. (A) Proliferation rates of the given epithelial cell subsets was analysed in PR/8 infected wt mice by FACS quantification of Ki67^+^ cells at the indicated time points pi. (B) Apoptosis of each EpCam^+^ subset was quantified by FACS (Annexin V^+^ proportions) at d7 post PR/8 infection and of non-infected wt mice. (C) Expression of Fgfr2b on EpiSPC at the given time points post PR/8 or mock infection was quantified by FACS and is given as MFI (median fluorescence intensity) of Fgfr2b ab minus MFI of matched isotype control. The proliferative response of the EpCam^+^ cell subsets was quantified by FACS at d7 pi in *Rosa26* ^*rtTA/+*^ *;tet(O)sFgfr2b/+* (D) *Rosa26* ^*rtTA/+*^ *;tet(O)Fgf10/+* mice (E) and *Fgf7* ^*-/-*^ mice (F) compared to non-dox-induced or wt littermates. Bar graphs represent means ± SD of n = 4–6 independent experiments; * *p*<0.05; **p<0.01; +dox, doxycycline food; -dox, normal diet.

Analyses of the proliferative response of various distal lung epithelial cell populations after PR/8 infection revealed that the EpiSPC population showed the highest proliferation capacity compared to the AEC and SAEC subsets between d7 to d14 after injury (Figs 2A and S4). Of note, comparison of EpiSPC and AEC proportions under homeostatic conditions and at d7 pi revealed an increase of the EpiSPC pool from 1.7 ± 0.3% to 5.7 ± 1.8% after IV- infection, whereas the high frequent AEC pool is reduced from 91.3 ± 1.8% to 84.7 ± 4.4%. Quantification of phosphatidylserine externalization by Annexin V expression of non-infected and PR/8 infected wt mice at d7 pi, a time point where apoptotic epithelial injury is most prominent in the lungs [8, 28], revealed that EpiSPC were resistant to IV-induced apoptosis, whereas the other EpCam^+^ populations showed high levels of apoptosis in response to infection (Fig 2B). These findings suggest that a damage-resistant cell population with high proliferative capacity is contained in the EpiSPC fraction, which might contribute to renewal of both bronchiolar and alveolar epithelial tissue [19].

Given that Fgf10 is an indispensable growth factor for EpiSPC outgrowth *ex vivo* and that genetic deletion of Fgf10 or of the Fgf10 receptor Fgfr2b results in failure of embryonic lung development [25], we speculated that this pathway might be reactivated to drive EpiSPC proliferation for epithelial regeneration after infectious injury in adult mice. In fact, Fgfr2b surface expression was significantly upregulated on EpiSPC in the course of severe PR/8 infection compared to mock-infected mice, most prominent at d7 post infection (pi) when the proliferative response was highly increased (Fig 2C). To decipher the functional role of Fgfr2b and its ligands Fgf10 and Fgf7 in this EpiSPC renewal response, transgenic mice with inducible overexpression of either soluble dominant negative Fgfr2b or overexpression of the ligand Fgf10, were PR/8-infected and proliferation of all EpCam^+^ subsets was quantified at d7 pi. Attenuation of Fgfr2b signaling by doxycycline induction of a dominant-negative Fgfr2b (scavenging soluble Fgf10) resulted in significant impairment of EpiSPC proliferation capacity compared to non-induced littermates, whereas AEC and SAEC revealed no or little, Fgfr2b-independent proliferation (Fig 2D). Similarly, the proliferating proportion of EpiSPC was significantly increased in mice with induced overexpression of Fgf10 at d7 pi, compared to non-induced litters (Fig 2E). Of note, *Fgf7* ^*-/-*^ mice exhibited only slightly but not significantly reduced EpiSPC proliferation in comparison to *Fgf7* ^*+/+*^ mice (Fig 2F). To verify that the Fgf10-Fgfr2b axis is indeed a key pathway in the epithelial regenerative response of the distal lung following IV-induced injury, we determined re-establishment of barrier function and outcome in doxycycline (dox)-induced versus non-induced *Rosa26* ^*rtTA/+*^ *;tet(O)sFgfr2b/+* mice. Blockade of Fgfr2b signaling resulted in significantly increased lung permeability (as determined by alveolar albumin leakage) during the repair phase at d14 pi (S5A Fig), indicating that this pathway is crucial for re-establishment of gas exchange function. Concomitantly, the surviving sFgfr2b overexpressing mice showed decreased body weight compared to controls during the regeneration phase at d11 to d20 pi, and did not fully regain weight until d21 (S5B Fig). Together, these findings demonstrate that IV infection induces activation of an Fgfr2b-dependent signaling pathway, which largely mediates the EpiSPC proliferative response and barrier repair after IV-induced injury.

### Highly pathogenic IV inhibit Fgfr2b-dependent renewal in EpiSPC by blockade of β-catenin mediated transcription

**Fig 3:**
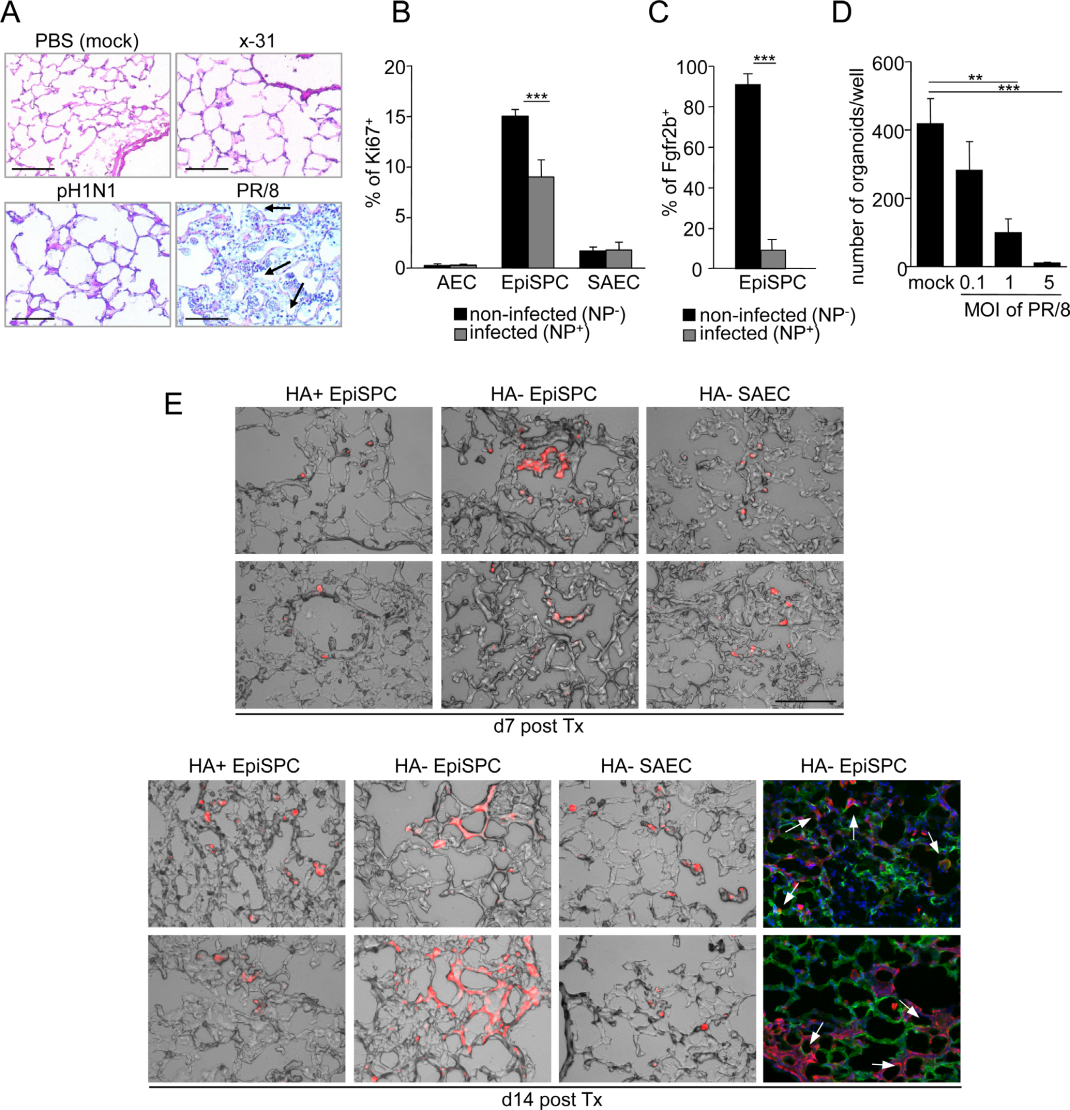
Influenza virus infected EpiSPC are impaired in their regenerative response due to restricted Fgfr2b expression. (A) Wt mice were infected with 500pfu of the indicated influenza virus strains, or mock-infected, and lung sections were stained with hematoxylin-eosin at d21 pi (arrows indicate areas of non-epithelialized tissue). (B) Infected and non-infected epithelial cell subsets of PR/8 infected wt mice were quantified by flow cytometry for their proliferative response. (C) Quantification of Fgfr2b expression in infected (NP^+^) and non-infected (NP^-^) EpiSPC by flow cytometry at d4 pi. (D) Flow-sorted EpiSPC were *ex vivo* infected with the indicated MOI of PR/8 and seeded in matrix for 3D cultures. At d6 of culture, the number of formed organoids was quantified. (E) Infected (hemagglutinin^+^; HA^+^) or non-infected (hemagglutinin^neg^; HA^-^) EpiSPC or control HA^-^ SAEC were flow-sorted from the lungs of PR/8-infected tdtomato^+^ mice at d4 pi for intrapulmonary transplantation into 7d PR/8-infected wt mice. Lung sections were obtained at d7 and d14 after transplantation. Representative micrographs show overlays of brightfield and red staining of tdtomato^+^ transplanted cells. Overlay of tdtomato^+^ transplanted cells (red) and the type I AEC cell marker T1α (green) is shown in the right panels (arrows indicate co-expression of T1α and tdtomato); bars = 100μm. Bar graphs represent means ± SD of n = 3–4 independent experiments; * *p*<0.05; **p<0.01; ***p<0.001; HA, hemagglutinin; Tx, transplantaion.

Given that the EpiSPC cell fraction harbored stem/progenitor cells crucial for Fgfr2b-drived lung repair and at the same time represented primary targets of high pathogenic PR/8 in the distal lung, we sought to address whether infection of these cells would result in an impaired renewal response. We therefore infected C57BL/6 mice using 500pfu of IV strains of increasing pathogenicity, i.e. x-31, pH1N1, and the highly pathogenic PR/8 [28, 29]. After 21 days, when mice had apparently recovered from IV infection, x-31 and pH1N1 infected mice showed a restored distal lung epithelial architecture, whereas mice infected with the highly pathogenic PR/8 still presented with thickened alveolar walls and incomplete re-epithelialization (Fig 3A), suggesting that IV of high pathogenicity impaired the regenerative response of the lung. Quantification of Ki67^+^ fractions in infected (NP^+^) versus non-infected (NP^neg^) EpiSPC within the same PR/8 infected lungs revealed that the proliferative response was lost in infected EpiSPC (Fig 3B), associated with loss of Fgfr2b upregulation in NP^+^ EpiSPC (Fig 3C). Finally, infection of flow-sorted EpiSPC *ex vivo* with increasing doses of PR/8 followed by organotypic culture resulted in significantly reduced formation of organospheres depending on the multiplicity of infection (MOI) applied (Figs 3D and S6). This was not due to infection-induced death of EpiSPC (as analysed by live/dead staining). Importantly and in line with these data, intratracheal transplantation of non-infected (viral hemagglutinin-neg; HA^neg^) EpiSPC, flow-sorted from the lungs of IV-infected tdtomato^+^ mice, into IV-infected wildtype mice, resulted in integration into and in *de novo* generation of distal lung tissue (including AEC I) between d7-d14 post transplantation, whereas infected (HA^+^) EpiSPC or SAEC showed incorporation into distal lung tissue, but only limited expansion and did not give rise to distal lung tissue (Figs 3E, S7A and S7B). These data indicate that EpiSPC indeed contain precursors of alveolar tissue *in vivo*, and IV infection of the EpiSPC niche results in defective tissue repair after IV-induced injury (Figs 3E and S7B). Of note, transplanted flow-sorted EpiSPC of non-infected tdtomato^+^ mice can be visualized at d14 post transplantation in the lung tissue of IV-infected wt mice, but do not expand to generate tissue *de novo* (S7B and S7C Fig), suggesting that factors expressed within the stem cell niche during IV-induced injury play a crucial role in early activation of quiescent EpiSPC for tissue repair (e.g. via IV-induced upregulation of the p63/krt5 regeneration program, Fig 1G).

**Fig 4:**
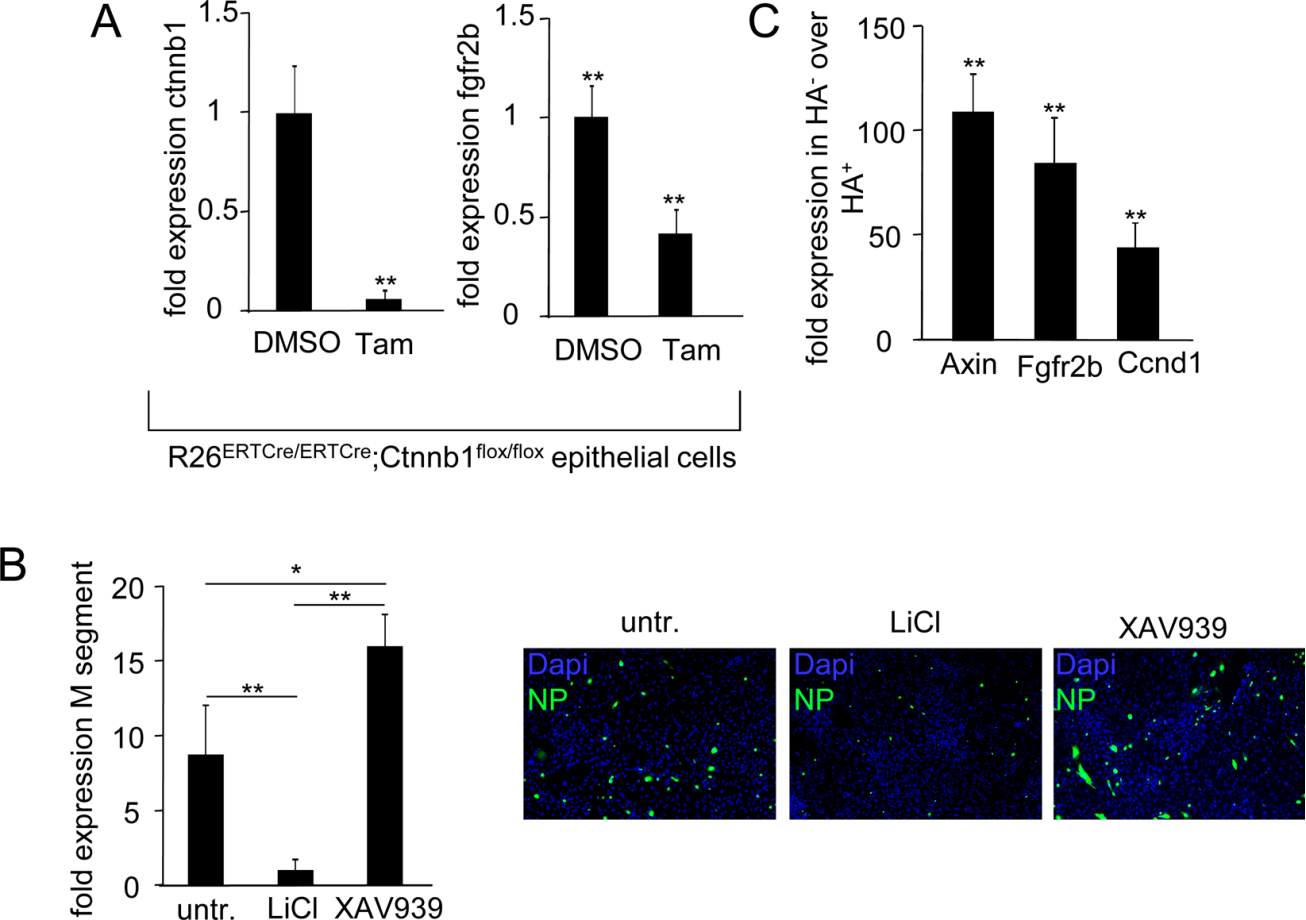
β-catenin dependent transcription mediates upregulation of *Fgfr2b* expression, which is inhibited in PR/8-infected, but not in non-infected lung epithelial cells. (A) EpCam^+^ lung epithelial cells derived from *Rosa26* ^*ERTCre/ERTCre*^ *;Ctnnb1* ^*flox/flox*^ mice were grown to confluency and treated with tamoxifen or DMSO control prior to infection with PR/8 (MOI = 0.1; 24h). mRNA expression of β-catenin (*Ctnnb1*) (left) or of *Fgfr2b* (right) was quantified and normalized to values of DMSO-treated control. (B) Wt distal lung epithelial cells in confluent culture were PR/8-infected (MOI 0.1) and treated with an activator (LiCl) or inhibitor (XAV939) of β-catenin signaling. Expression of the viral M segment was quantified at 16 h pi and normalized to LiCl-treated cultures (left). The right plot shows representative photomicrographs of these cultures stained for IV nucleoprotein (NP) after 6 h of PR/8 infection. (C) Wt mice were infected with PR/8 for 7d and infected (IV hemagglutinin^+^, HA^+^) vs. non-infected (HA-) EpCam^+^ cells were flow-sorted. Expression of the β-catenin-dependent transcripts *Axin2*, *Fgfr2b*, and *Ccnd1* was quantified in HA- cells and normalized to values from HA^+^ cells. All bar graphs represent means ± SD of n = 3–4 independent experiments; * *p*<0.05; **p<0.01; Tam, tamoxifen.

We next addressed the putative mechanism of inhibition of Fgfr2b upregulation in infected EpiSPC. Previous data revealed that Fgfr2b expression is dependent on Wnt/β-catenin signaling in the developing lung [31]. Indeed, conditional knockout of β-catenin by tamoxifen treatment in adult distal lung epithelial cells from *Rosa26* ^*ERTCre/ERTCre*^ *;Ctnnb1* ^*flox/flox*^ mice grown *ex vivo* (Fig 4A, left) resulted in impaired upregulation of *fgfr2b* mRNA expression after PR/8 infection (Fig 4A, right). Recent data suggest that β-catenin is involved in expression of interferon-dependent genes and that IV block β-catenin transcriptional activity *in vitro* as part of an antiviral escape strategy [32]. In fact, activation of the Wnt/β-catenin pathway by LiCl resulted in widely reduced IV replication in *ex vivo* cultured distal lung epithelial cells, whereas inhibition increased replication, as demonstrated by immunofluorescence and quantification of the viral *m segment* expression (Fig 4B). Concomitantly, expression of the β-catenin target genes *Axin* and *Ccnd1*, and of *Fgfr2b*, was reduced by ∼50 to 100-fold in infected (HA^+^) compared to non-infected (HA^neg^) EpiSPC flow-sorted from PR/8-challenged mice at d7 pi (Fig 4C). These data indicate that EpiSPC tropism of IV represents a key factor of pathogenicity. IV interfere with β-catenin-dependent gene transcription in infected EpiSPC, likely to escape β-catenin anti-viral properties, which results in impaired Fgfr2b expression and reduced renewal capacity in infected EpiSPC.

### Exogenous application of excess Fgf10 compensates impaired regeneration of the lung epithelial barrier and improves outcome after severe IV infection

**Fig 5:**
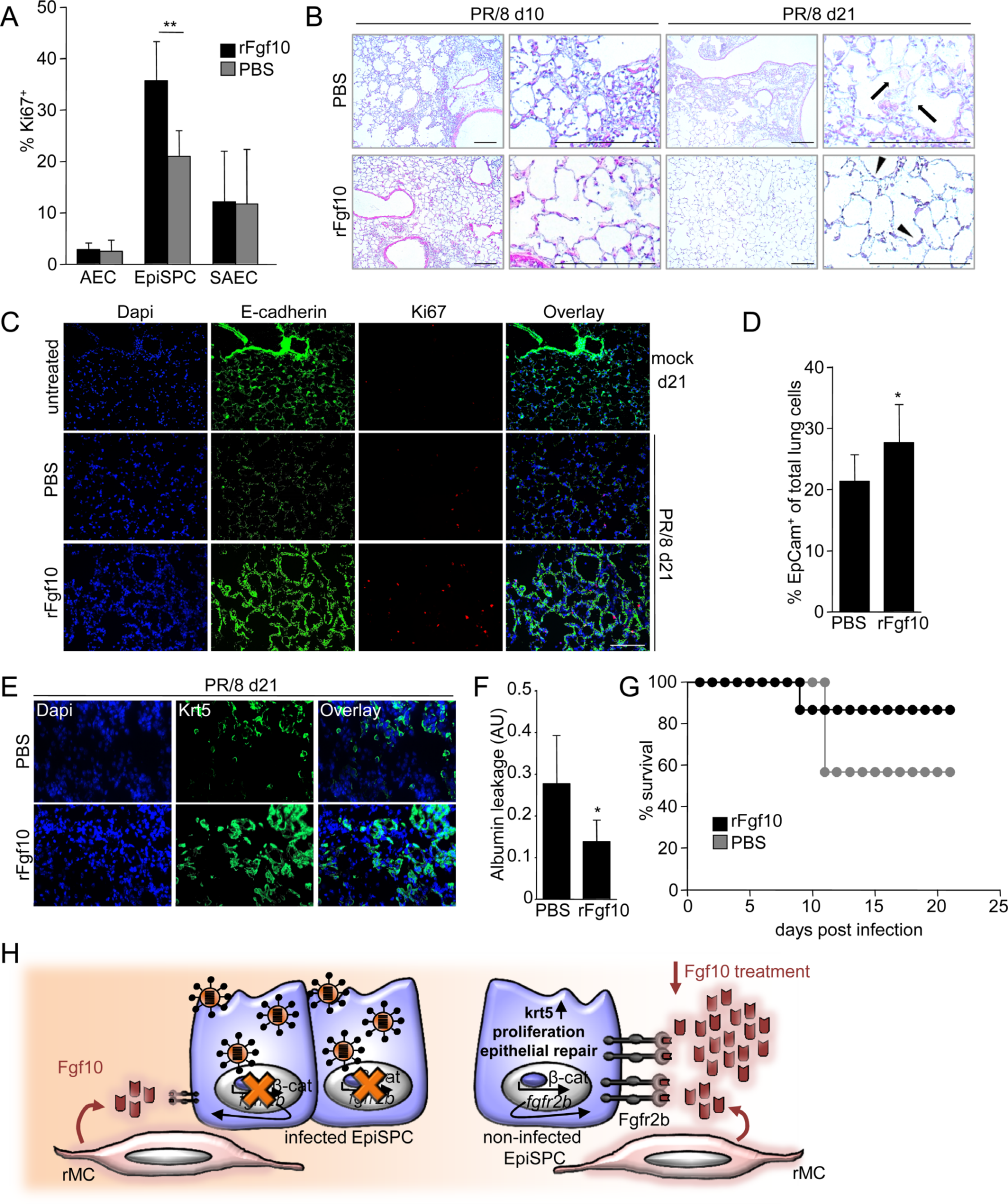
Therapeutic treatment with recombinant Fgf10 improves influenza virus-induced lung injury and improves re-epithelialization and barrier repair. Wt mice were infected with PR/8 and treated with a single dose of either 5μg recombinant Fgf10 (rFgf10) or diluent (PBS^-/-^) at d6 pi. (A) The proliferative response of EpCam^+^ epithelial cell subsets was determined by flow cytometry at d7 pi. (B) Lung sections were stained with hematoxylin-eosin at d10 and d21 pi. Arrows depict non-epithelialized alveolar tissue; arrowheads depict areas of ongoing re-epithelialization. (C) Immunofluorescence staining of lung sections for E-cadherin (green), Ki67 (red), and Dapi (blue) at d21 pi. The top row shows lung tissue from mock-infected, untreated mice at d21. (D) Quantification of total lung epithelial cells (EpCam^+^) in lung homogenates at d14 pi. (E) Lung sections were stained for krt5 (green) and Dapi (blue) at d21 pi. (F) Lung barrier function was analysed by quantification of alveolar leakage of FITC-labeled albumin at d14 pi. Values are given in arbitrary units (AU) and represent ratios of FITC fluorescence in BALF and serum. (G) Survival of n = 8 mice per treatment group was analysed until d21 pi. Bar graphs represent means ± SD of n = 5–6 independent experiments; * *p*<0.05; **p<0.01. Photomicrographs are representative for n = 3–4 independent experiments; bars = 200 μm. (H) Summary: IV with high pathogenicity infect a substantial fraction of EpiSPC, resulting in inhibition of β-catenin-dependent Fgfr2b upregulation and impaired epithelial repair mediated by rMC-expressed Fgf10. Therapeutic application of excess Fgf10 antagonizes IV-induced regeneration failure by engagement of non-infected, Fgfr2b^high^ EpiSPC.

To evaluate whether alveolar deposition of excess Fgf10 would counteract the impaired Fgfr2b-mediated renewal response in IV infected mice by increased recruitment of the non-infected Fgfr2b^high^ EpiSPC, we applied recombinant Fgf10 or PBS to IV-infected C57BL/6 mice at d6 pi. Indeed, Fgf10 treatment resulted in significantly increased proliferation of EpiSPC compared to PBS-treated mice at d7 pi (Fig 5A). IV-infected mice showed a severely disturbed lung architecture with distinct cellular infiltrates, areas of extensive atelectasis and loss of epithelial cells at d10 pi, which was partially reverted by Fgf10 treatment (Fig 5B, left). By d21, Fgf10-treated mice revealed an almost normal lung structure with re-epithelialized bronchioli and alveoli (Fig 5B, right, arrowheads), whereas PBS-treated controls still presented with areas of atelectasis, cellular infiltrates and epithelial injury, indicating failure of epithelial renewal and persisting injury-associated inflammatory responses (Fig 5B, right, arrows). To verify that Fgf10 indeed impacted re-establishment of bronchiolar and alveolar epithelial structures, E-cadherin stainings of lung sections were performed and revealed that Fgf10 treatment resulted in complete re-establishment of the epithelium at d21 pi, associated with increased numbers of Ki67^+^ proliferating cells, whereas PBS-treated mice showed only partial epithelial renewal (Fig 5C). These findings were confirmed by quantification of the total numbers of EpCam^+^ cells in these treatment groups (Fig 5D). Of note, Fgf10-treated murine lungs showed increased expression of krt5, a marker of stem cell-induced repair, at d21 pi (Fig 5E) [16, 17]. Finally, Fgf10-mediated epithelial repair resulted in improved barrier function at d14 pi, and improved survival until d21 compared to controls (Fig 5F and 5G), highlighting the therapeutic potential of Fgf10 to improve EpiSPC-dependent epithelial regeneration.

## Discussion

Repair of the injured lung epithelium including structural and functional re-establishment of alveolar barrier function after severe IV pneumonia is crucial for recovery and survival. In this study, we demonstrate that a fraction of distal lung epithelial cells phenotyped as EpCam^high^α6β4^high^CD24^low^Sca-1^high^CD200^+^ EpiSPC drive epithelial renewal processes involving Fgf10/Fgfr2b-mediated signaling. Of note, the impaired alveolar regeneration after infection with highly pathogenic IV observed in mice and reported in humans [3] was associated with increased viral infection rates of EpiSPC in the distal lung compared to AEC and SAEC, and IV-induced inhibition of β-catenin-dependent gene transcription impaired regenerative Fgfr2b-signaling in these progenitor cells. Whereas transplantation of non-infected EpiSPC isolated from murine lungs, resulted in integration into lung tissue and *de novo* generation of distal lung epithelium including AEC I, previously infected EpiSPC did not give rise to lung tissue in the distal lung. These data highlight that tropism of IV to subsets of the lung stem cell niche may be a crucial determinant of IV pathogenicity resulting in severe impairment of Fgfr2b-mediated lung regeneration, and likely persistent failure of barrier function and worsened outcome.

Regeneration of the epithelial compartment of the distal lung was shown to involve different stem/progenitor cell populations, including p63^+^krt5^+^ lineage-negative, β4^+^ epithelial progenitors or distal airway stem cells (DASC^p63/krt5^) [16, 17], α6β4^high^ alveolar cells, and more lineage-committed CC10^+^ or SPC^+^ populations [14, 17, 33, 34]. Our data show that the EpiSPC population phenotyped as EpCam^high^α6β4^high^CD24^low^Sca-1^+^CD200^+^ [19, 35] contained cells with stem characteristics as verified by organoid outgrowth in 3D cultures, and clonogenic potential in presence of growth factors including Fgf10. Furthermore, EpiSPC gave rise to cells expressing markers of terminally differentiated airway and alveolar epithelium in matrigel, suggesting that they are precursors of bronchiolar and alveolar epithelium. EpiSPC displayed high proliferation capacity after bronchio-alveolar injury caused by IV infection, as opposed to other distal lung epithelial cell populations. This renewal response was associated with strong induction of the p63/krt5 regeneration program, found to be crucial for distal lung repair [17, 36]. Furthermore, Fgf10-treated mice increased krt5 expression in their lungs, suggesting that the cells we identify as EpiSPC contribute to the p63/krt5 pool, and that expansion or generation of krt5^+^ cells is dependent on Fgf10. Conflicting data exist on the capacity of lineage-committed cells to be progenitors of differentiated distal lung cells [17, 37, 38]. Zheng et al suggested that most of the newly induced p63^+^ cells in the IV-damaged distal lung compartment might be derived from CC10^+^ cells, whereas a recent report defined them as lineage-negative [17, 39], highlighting that the contribution of different stem/progenitor populations to alveolar repair may be injury-specific, dependent on the region and extent of injury, and on microenvironmental factors regulated in the context of defined types of damage. With respect to recent data highlighting the AEC II pool as stem cell niche of the alveolar epithelium, we found that the EpCam^low^α6^low^ AEC II fraction proliferated only to a limited extent after IV infection *in vivo*. However, our data do not fully exclude contribution of an AEC II progenitor to the alveolar regeneration process, as demonstrated for bleomycin-induced damage [33], particularly as AECII constitute a highly abundant cell population of the lung, and even a small proliferating AECII fraction could still contribute to epithelial repair.

Clearly, EpiSPC proliferation after IV-induced injury largely depended on Fgfr2b and its ligand Fgf10, as demonstrated by use of *Rosa26* ^*rtTA/+*^ *;tet(O)sFgfr2b/+*, and *Rosa26* ^*rtTA/+*^ *;tet(O)Fgf10/+* mice, whereas Fgf7 did not substantially contribute. Given that *fgf7* ^*-/-*^ mice are viable and do not display gross lung abnormalities as compared to *Fgf10* ^*-/-*^ and *Fgfr2b* ^*-/-*^ mice [25], and given that organoid formation from EpiSPC *ex vivo* is strictly dependent on Fgf10 but does not require Fgf7 [30], we conclude that Fgf10 rather than Fgf7 mediates EpiSPC renewal, although both ligands share the same receptor. A recent report highlights different functions of these ligands with respect to Fgfr2b processing and recycling [40], suggesting that the prolonged proliferative response observed after exogenous Fgf10 application at d21 pi might be associated with Fgf10-induced maintenance of Fgfr2b expression on EpiSPC. With respect to cellular origin of Fgfr2b ligands within the stem/progenitor cell niche, an EpCam^neg^Sca-1^high^ cell population of non-leukocyte and non-endothelial lineage [24] was found to be the primary source and supported for lung organoid formation in 3D culture. The lung mesenchyme is known to be the cellular origin of Fgf10 during lung organogenesis and postnatally [23, 24], and therefore represents a key orchestrator of the EpiSPC niche. Cellular responses of Fgf10 in the developing lung include epithelial progenitor cell maintenance and prevention of epithelial differentiation [41]. Our data confirm a central role of Fgf10 in survival and proliferation in adult lung EpiSPC *ex vivo* and *in vivo*, and demonstrate that neutralization of Fgf10 in EpiSPC-mesenchymal cell co-cultures results in inhibition of sphere outgrowth at a very early stage.

Canonical β-catenin signaling was found to induce expression of the *Fgfr2b* gene in the developing lung epithelium [31]. A key finding reported here is that IV infect EpiSPC and interfere with β-catenin-dependent gene transcription of Fgfr2b, resulting loss of Fgfr2b upregulation in the infected fraction of EpiSPC, which are thus unable to proliferate to promote repair. It has been recently demonstrated that β-catenin is indispensable for expression of type I interferon in response to IV infection in different cell lines. Many viruses have evolved gene products during co-evolution with their hosts by which they can induce and control various responses of their host cell, in particular early innate immune pathways. Mechanistically, a direct interaction of the viral NS1 protein, a potent antagonist of host innate antiviral responses [42], with the Wnt receptor *frizzled* upstream of canonical β-catenin signaling was discussed [43]. More recent publications suggest an interaction of Influenza or Sendai Virus-induced host cell components of the NF-κB pathway or of IRF3, respectively, with nuclear β-catenin to repress β-catenin-dependent gene transcription [32, 44]. Additionally, another report suggests that expression of the pandemic 1918 IV polymerase subunit PB2 increases virulence by inhibition of the Wnt signaling cascade which impacts on regeneration of the inflamed lung tissue [45]. Our own data clearly support the concept that the canonical β-catenin pathway is anti-viral, as demonstrated in studies using primary distal lung epithelial cells *ex vivo* infected with IV in presence of a β-catenin activator or inhibitor. This suggests that inhibition of β-catenin-dependent gene transcription is a conserved strategy of viral immune escape, which additionally results in blockade of renewal programs in EpiSPC. Our data furthermore provide a comprehensive, FACS-based quantification of infection rates of various lung epithelial cell compartments of the distal murine lung, and particularly indicate that the extent of EpiSPC infection by different IV strains represents an important, previously undefined factor of viral pathogenicity. In addition, the finding that EpiSPC as opposed to differentiated epithelium [10] are not subjected to infection-associated apoptosis but survive (likely due to constitutive expression of maintenance/renewal-associated survival pathways), raises the question whether viral ´imprinting´ will cause changes of transcriptional or epigenetic programs of stem cell plasticity resulting in long-term epithelial dysfunction.

Altogether, we provide evidence that pathogens such as IV severely affect the progenitor cell-mediated, Fgfr2b-dependent repair of the distal lung epithelium, and that intratracheal treatment of pathogen-injured lungs with excess Fgf10, to recruit the non-infected, Fgfr2b^high^ EpiSPC fraction, promoted epithelial renewal without inducing aberrant repair [46] at the dose used. Fgf10 might therefore represent a putative treatment option to foster organ repair and re-establish gas exchange function after IV-induced and possibly other forms of ARDS.

## Materials and Methods

### Mice

Wildtype C57BL/6 mice were purchased from Charles River Laboratories. *CMV-Cre* mice [47] were crossed with *rtTA* ^*flox*^ mice [48] to generate mice expressing *rtTA* under the ubiquitous *Rosa26* promoter. This constitutive *Rosa26* ^*rtTA/rtTA*^ mouse line was then crossed with *tet(O)sFgfr2b/+* or *tet(O)Fgf10/+* responder lines to generate *Rosa26* ^*rtTA/+*^ *;tet(O)sFgfr2b/+* and *Rosa26* ^*rtTA/+*^ *;tet(O)Fgf10/+* double heterozygous animals on a mixed genetic background, allowing ubiquitous expression of dominant-negative soluble Fgfr2b [49, 50] or of Fgf10 [18, 46] by administration of doxycycline-containing normal rodent diet with 0.0625% doxycycline (Harlan Teklad). Mice were genotyped as described previously [46, 50–52]. *Fgf7* ^*-/-*^ mice were obtained from Jackson Laboratory and backcrossed for several generations on a C57BL/6 background (strain #4161). B6.129(Cg)-*Gt(ROSA)26Sor* ^*tm4(ACTB-tdTomato*,*-EGFP)Luo*^/J (mTmG) mice in C57BL/6 genetic background, a tamoxifen-responsive driver mouse line, and *Ctnnb1* ^*flox/flox*^ mice were obtained from Jackson Laboratory (strains #7676, #3309 and #4152) and the latter bred to generate homozygous *Rosa26* ^*ERTCre/ERTCre*^ *;Ctnnb1* ^*flox/flox*^ mice on a C57BL/6 background allowing induction of a β-catenin knockout by application of tamoxifen. Mice were housed under pathogen-free conditions and experiments were performed according to the regional institutions´ guidelines.

### Reagents

The following antibodies were used for flow cytometric analyses, cell sorting or immunofluorescence: CD49f PE or Pacific Blue (clone: GoH3), CD326 (EpCam) APC-Cy7 or FITC (clone G8.8), CD24 PE-Cy7 (clone: M1/69), Ly-6A/E (Sca-1) PerCP/Cy5.5 or Pacific Blue (clone: D7), CD31 Alexa fluor 488 or PE (clone: MEC13.3), CD45 FITC or APC-Cy7 (clone: 30-F11), CD200 PE (clone: OX-90), T1α/podoplanin APC (clone: 8.1.1.) and corresponding isotype controls syrian hamster IgG (clone SHG-1); all Biolegend. Influenza A virus nucleoprotein (NP) FITC (clone: 431, Abcam), Fgfr2b (clone: 133730) and corresponding isotype control IgG2a (clone: 54447, both R&D Systems), p63 (Life Span Biotechnology), CC10 (clones T-18 and S-20) and isotype-matched normal goat IgG (Santa Cruz Biotechnology), CD104 Alexa fluor 647 (clone: 346-11A, AbD SeroTec), Ki67 FITC or PE (clone: B56) and corresponding isotype control IgG1κ FITC or PE (clone: MOPC-21, both BD Bioscience), Annexin V Alexa fluor 647 (Invitrogen), E-cadherin (clone: DECMA-1, Abcam), p63 Alexa fluor 555 (clone: P51A, Bioss), Podoplanin (clone: RTD4E10, Abcam) or corresponding isotype control syrian hamster IgG (clone: SHG-1, Abcam), beta IV tubulin (clone: ONS.1A6) and corresponding isotype control IgG1 (clone: CT6, both Abcam), cytokeratin 5 FITC (Bioss), Uteroglobin (clone: EPR12008, abcam) and isotype-matched monoclonal rabbit IgG (abcam), purified Ki67 (Thermo Scientific), purified pro-surfactant protein C (Millipore), biotinylated mucin 5AC (clone: 45M1, Abcam), anti-influenza NP (clone: 1331, Meridian Life Science). Secondary antibodies used were anti-Streptavidin APC (Becton Dickinson), anti-rabbit Alexa fluor 488/555, anti-goat Alexa fluor 647, anti-mouse Alexa fluor 555/647, anti-rat Alexa fluor 488/647, anti-hamster Alexa fluor 488 (all Molecular Probes). Magnetic separation was performed using biotinylated rat anti-mouse CD45, CD16/32 and CD31 mAb (BD Bioscience, Pharmingen). For *ex vivo* neutralization assays anti-fgf10 (clone: C-17) or normal goat IgG (Santa Cruz Biotechnology) were used at a concentration of 5μg/ml. For flow cytometric analyses, cells were routinely stained with 7-AAD (Biolegend) or fixable live/dead stain reagents (Molecular Probes) for dead cell exclusion.

### *In vivo* treatment protocols

Mice were anaesthesized and intratracheally inoculated with 500pfu (plaque forming units) of A/PR/8/34 (H1N1, mouse-adapted), A/x-31 (H3N2), or A/Hamburg/5/09 (pandemic H1N1), grown and quantified in Madin Darby Canine Kidney (MDCK) cells (obtained from American Type Culture Collection) and diluted in a total volume of 70 μl in sterile PBS^-/-^. In the treatment approach, 5 μg recombinant Fgf10 (R&D Systems) dissolved in sterile PBS^-/-^ or PBS^-/-^ alone were intratracheally applied to IV infected mice. Venous blood and BALF were collected as described previously [53]. Lung permeability was determined by i.v. injection of 100 μl FITC-labeled albumin (Sigma-Aldrich) and quantification of FITC fluorescence ratios in BALF and serum with a fluorescence reader (FLX800, Bio-Tek instruments) as described elsewhere [28]. In selected experiments, 20,000 flow-sorted HA^+^ or HA^neg^ EpiSPC from IV infected mTmG mice were intratracheally applied into IV infected wt mice at d7 pi. Virus titers from BALF were determined by immunohistochemistry-based plaque assay on confluent MDCK cells in 6-well plates in duplicates as previously described [54]. Infected mice were monitored 1–3 times per day and a morbidity score was calculated from weight loss, general appearance, breathing frequency/dyspnea, and body temperature. Mice with a score ≥ 20 were moribund, sacrificed and classified as dead in mortality studies.

### Isolation of murine distal lung cells

Lung homogenates of distal lung cell suspensions were obtained by instillation of dispase (BD Biosciences) and 0.5 ml low-melting agarose (Sigma) through the trachea into the HBSS (Gibco) perfused lung, followed by incubation in dispase for 40 min as previously described [55]. After gelling of the agarose and removal of the agarose-filled trachea and proximal bronchial tree, the lung was homogenized (GentleMACS, MACS Miltenyi Biotech) in DMEM/2.5% HEPES with 0.01% DNase (Serva) and filtered through 100μm and 40μm nylon filters. Cell suspensions were incubated with biotinylated rat anti-mouse CD45, CD16/32 and CD31 mAb for 30 min at 37°C followed by incubation with biotin-binding magnetic beads and magnetic separation to deplete leukocytes and endothelial cells prior to flow cytometric analysis and cell sorting or to further culture.

### Flow cytometry and cell sorting

Multicolor flow cytometry or high speed cell sorting was performed with an LSR Fortessa or an Aria III cell sorter using DIVA software (BD Bioscience). For analytical measurements 1–5 x 10^5^ cells were freshly stained with fluorochrome-labeled antibodies for 20 min at 4°C in BD FACS buffer. For intracellular stainings (NP, proSPC), permeabilization of cells was achieved by previous incubation with 0.2% saponin in PBS^-/-^ for 15 min at 4°C, followed by incubation with anti-NP FITC, anti-proSPC, or respective isotype control mAbs for 20 min at 4°C. When non-labeled primary mAb were used, a fluorescent labeled secondary Ab was added and incubated for 20 min at 4°C in FACS buffer. Doublets and dead cells were routinely excluded from the analyses (the latter by using 7AAD). Annexin V staining was performed on fresh, non-permeabilized cells. Prior to antibody incubation, cells were washed and resuspended in Annexin V buffer (BD Bioscience) and incubated with Annexin V 647 and further mAbs in Annexin V buffer for 20 min at 4°C. The stained cells were washed and resuspended in Annexin V buffer. Cell sorting was performed with an 85 or 100μm nozzle. Single cell sorting was performed using the automated cell deposition unit (ACDU) with a 24-well plate and 12mm cell culture inserts (0.4μm pore size, Millipore). Purity of flow-sorted cells was always > 95%.

### Culture of flow-sorted lung cells in matrix and clonogenic assays

Mono- or co-culture of EpiSPC and rMC was performed as described previously [30]. In brief, flow sorted cells were counted, resuspended and mixed with growth factor reduced matrix (BD Biosciences) diluted with EpiSPC medium (α-MEM, 10% FCS, 1x pen/strep, 1x insulin/transferrin/selenium, 2mM L-glutamine, 0.0002% heparin) at a 1:1 ratio. Cell suspensions were seeded in 12mm cell culture inserts (0.4μm pore size, Millipore) in a 24-well plate and incubated for 5 min at 37°C, 5% CO_2_ to allow gelling. EpiSPC medium was then added to the bottom wells of the plate. In selected experiments, 50ng/ml recombinant Fgf10 and 30ng/ml recombinant Hgf (both R&D systems) or anti-Fgf10 or control Ab were added to the medium at day 2 of culture. For matrix digestion a preheated dispase/collagenase I (Boehringer, Gibco) mixture (3 mg/ml) was added and a single cell suspension was obtained for re-seeding or single-cell sorting. Images were taken with a DM IL LED microscope and a corresponding camera MC170 HD (Leica).

### Immunofluorescence and immunohistochemistry

To obtain lung cryosections, lungs were perfused with HBSS and filled with 1.5ml TissueTek (Sakura) mixed with PBS^-/-^ at a 1:1 ratio as described [56]. Lungs were removed, snap-frozen and 4–10 μm sections were prepared using a Leica cryotome. In selected experiments, lungs were filled with a TissueTek/PBS^-/-^ mixture containing 1% paraformaldehyde and were incubated in 1% paraformaldehyde after removal. Lung cryosections were stained with Hematoxylin-Eosin or fixed with 4% paraformaldehyde for 20 min, blocked with 0.05% Tween 20, 5% BSA, 5% horse serum in PBS^-/-^ for 30 min and stained with fluorochrome-labeled mAb diluted in PBS^-/-^, 0.1% BSA, 0.2% Triton X-100 for 2 h. After washing, secondary mAbs were added for 2 h, followed by mounting with Dapi containing mounting medium (Vectashield, Vector Labs). Epithelial or mesenchymal cells cultured in chamber slides (Nunc) were fixed in a 1:1 ratio of cold acetone/methanol for 5 min and blocked with 3% BSA in PBS for 30 min prior to staining. Cytospins were additionally stained with Pappenheim stain. Analysis was performed with a Leica DM 2000 or with the Evos Fl Auto (Invitrogen) microscope.

### *Ex vivo* infection of primary cells

Primary murine distal lung cells contained >90% epithelial cells as determined by FACS. Cells were grown in 24-well plates (Greiner) or in chamber slides (Nunc) in DMEM enriched with HEPES, L-Glutamine, FCS, and pen/strep, until confluency and infected with PR/8 at the indicated MOI, as described previously [55]. PR/8 was diluted in PBS^-/-^ containing BSA and pen/strep and added to the cells for 1 h, until the inoculum was removed and changed to infection medium (DMEM supplemented with BSA, pen/strep, L-Glutamine and trypsin) for further incubation. For inhibition of β-catenin, isolated distal lung epithelial cells of *Rosa26* ^*ERTCre/ERTCre*^ *;Ctnnb1* ^*flox/flox*^ mice were treated with 1 μM tamoxifen (Sigma-Aldrich) for 24 hours prior to PR/8 infection. Wildtype cells were treated with either 50 mM LiCl (Abcam) or 10 μM XAV939 (Abcam) directly after PR/8 infection, followed by RNA extraction or immunofluorescence staining. For infection of FACS-sorted lung cells (AEC, EpiSPC, SAEC), the cells were counted, seeded in wells or infected in tubes for 1 hour with the given virus strain and MOI, and were either seeded in matrix, or further incubated at 37°C, 5% CO_2_ with EpiSPC medium and processed for FACS analysis at the given time point pi.

### Quantitative real-time PCR

RNA from sorted or cultivated cells was isolated using RNeasy Kit (Qiagen) according to manufacturer's manual. cDNA synthesis was performed, as described previously [56] or with RiboSPIA kit (NuGen) according to manual. Actin or ribosomal protein subunit S-18 (RPS-18) expression served as normalization controls for the qRT-PCR, and the reactions were performed with SYBR green I (Invitrogen) in the AB Step one plus Detection System (Applied Bioscience). The following intron spanning primers were used: *Actin* (FP, 5′-ACCCTAAGGCCAACCGTGA-3′; RP, 5′-CAGAGGCATACAGGGACAGCA-3′), *Rps-18* (FP, 5’- CCGCCATGTCTCTAGTGATCC-3′; RP, 5’- TTGGTGAGGTCGATGTCTGC-3′), *p63* (FP, 5’-CAAAGAACGGCGATGGTACG-3′; RP 5’-CCTCTCACTGGTAGGTACAGC-3′), *Krt5* (FP, 5’-CCTTCGAAACACCAAGCACG-3′; RP 5’-AGGTTGGCACACTGCTTCTT-3′), β-*tubb* (FP, 5’-CCACCACCATGCGGGAAA-3′; RP, 5’-CTGATGACCTCCCAGAACTTG-3′), *Fgf10* (FP, 5’-CCATGAACAAGAAGGGGAAA-3′; RP 5’-CCATTGTGCTGCCAGTTAAA-3′), *Fgf7* (FP, 5’- TCGCACCCAGTGGTACCTG-3′; RP, 5’- ACTGCCACGGTCCTGATTTC-3’), *Axin2 (FP*, 5’-AAGCCCCATAGTGCCCAAAG-3′; RP, 5’-GGGTCCTGGGTAAATGGGTG-3′), *Fgfr2b* (FP, 5’- AAGAGGACCAGGGATTGGCA-3′; RP, 5’- GTACGGTGCTCCTTCTGGTTC-3′), *Ctnnb1* (FP, 5’- ACTTGCCACACGTGCAATTC-3′; RP, 5’-ATGGTGCGTACAATGGCAGA-3′), *Ccnd1* (FP, 5’- GCGTACCCTGACACCAAT-3′; RP, 5’- GGTCTCCTCCGTCTTGAG-3′), *Pdpn* (FP, 5’-CCCCAATAGAGATAATGCAGGGG-3′; RP, 5’-GCCAATGGCTAACAAGACGC-3′), *Influenza Virus M segment* (5’-GGACTGCAGCGTAGACGC-3′; 5’ CATCCTGTTGTATATGAG-3′; 5’-CATTCTGTTGTATATGAG-3′). The relative gene abundance compared to the reference gene *Actin* or *Rps-18*) was calculated as dCt value (Ct_reference_−Ct_target_). Data are presented as dCT, ddCt (dCt_reference_—dCt_target_) or fold change of gene expression (2^ddCt^).

### Ethics statement

Animal experiments performed at the UGMLC were approved by the regional authorities of the State of Hesse (Regierungspräsidium Giessen; reference numbers 100–2012, 48–2013, 26–2013, 09–2009) and conducted according to the legal regulations of the German federal animal protection law (Tierschutzgesetz).

### Statistics

All data are given as mean ± SD. Statistical significance between 2 groups was estimated using the unpaired Student’s *t* test or ANOVA and post-hoc Tukey for comparison of 3 groups and calculated with GraphPadPrism. A *p* value less than 0.05 was considered significant.

## Supporting Information

S1 Fig Tropism of different IV strains to AEC, EpiSPC and SAEC *ex vivo*. Flow sorted AEC, EpiSPC and SAEC were seeded in culture plates to equal densities and infected *ex vivo* with PR8, pH1N1 or H3N2 (x-31) at MOI 2, respectively. After 6h, infection rates were quantified by flow cytometric analysis by gating on nucleoprotein (NP)-positive fractions. Bar graphs show mean values ± SD for n = 3 individual experiments; * *p*<0.05; **p<0.01; ***p<0.001.
(TIF)

S2 Fig EpiSPC show clonal expansion and organoid outgrowth in 3D cultures supplemented with growth factors. (A) AEC, SAEC and EpiSPC flow-sorted from wt mice were cultured in 3D matrix with or without (w/o) growth factor (GF) supplementation. (B) EpiSPC were single cell sorted into matrix using the BD ACDU (automated cell deposition unit), resulting in clonal expansion and organoid outgrowth. After one week of culture, cell suspensions were prepared from the organoids and single cells were reseeded. This was repeated for at least 6 times, demonstrating high clonal potential of EpiSPC. (C) Single cell suspensions of flow-sorted EpiSPC derived from tdtomato and wt mice were mixed and cultured in matrix, resulting in generation of either tdtomato^+^ or tdtomato^neg^ organoids.
(TIF)

S3 Fig Sca-1^high^ lung resident mesenchymal cells drive EpiSPC lung-like organoid formation and cell differentiation in an Fgf10-dependent manner. (A) Gating strategy of lung tissue for the separation of endothelial cells (R1), leukocytes (R2), epithelial cells (R3) and Sca-1^high^ mesenchymal cells (R4). mRNA expression (ΔCT to housekeeping gene) of Fgf10 and Fgf7 in flow-sorted R1-R4 populations from mock or PR/8 infected wt mice. (B) Matrix co-culture of resident mesenchymal cells (rMC) and EpiSPC without growth factor supplementation results in generation of lung-like structures at d16 of culture. The left photomicrograph shows mono-cultured EpiSPC in the presence of growth factors. (C) Co-culture of EpiSPC with CD31^+^ endothelial (R1) cells or CD45^+^ leukocytes (R2) instead of rMC does not result in lung-like organoid formation at d10 of culture. (D) Organoid outgrowth in non-GF-supplemented rMC-EpiSPC co-cultures, is inhibited in presence of a neutralizing anti-Fgf10 ab, but not with corresponding isotype IgG ab, at d5 of culture. (E) After 10d of co-culture (rMC) versus GF-supplemented mono-culture (-), organoids were isolated and mRNA expression of marker genes of alveolar (T1α/podoplanin) and airway (β-tubulin) differentiation was quantified. Values are normalized to freshly flow-sorted EpiSPC, respectively. All bar graphs represent means ± SD of n = 3 independent experiments; * *p*<0.05; **p<0.01; ***p<0.001. Significances in (A) refer to the PR8 or mock group of R4, respectively. Photomicrographs are representative for n = 3 independent experiments.
(TIF)

S4 Fig Representative FACS plot of proliferation analysis in EpCam^high^α6^high^CD24^low^ cells. EpCam^high^α6^high^CD24^low^ cells were subgated for the Ki67^+^ fraction. Gates were set according to controls containing fluorochrome-labeled isotype control.
(TIF)

S5 Fig Inhibition of Fgfr2b signalling results in impaired re-establishment of lung barrier function and worsened outcome after PR/8 infection. Doxycycline-induced or non-induced *Rosa26* ^*rtTA/+*^ *;tet(O)sFgfr2b/+* mice were infected with PR/8 and lung barrier function was analyzed by quantification of alveolar leakage of FITC-labeled albumin at d14 pi. Values are given in arbitrary units (AU) and represent ratios of FITC fluorescence in BALF and serum and are derived from n = 7 independent experiments (A). Body weight (B) of doxycycline-induced vs. non-induced *Rosa26* ^*rtTA/+*^ *;tet(O)sFgfr2b/+ mice* (n = 8, respectively) after PR/8 infection were analyzed until d21 pi. Bar graphs represent means ± SD; * *p*<0.05; +dox, doxycycline food; -dox, normal diet.
(TIF)

S6 Fig FACS quantification of infected EpiSPC after *ex vivo* infection. Flow-sorted EpiSPC in suspension were PR/8 infected for 8h at continuous rotation (bottom) or were left un-infected (top). The infected fractions were quantified by FACS using IV nucleoprotein (NP) staining (∼44% at MOI = 5).
(TIF)

S7 Fig Detection of and tissue generation from intratracheally transplanted tdtomato^+^ EpiSPC in the distal lung of PR/8-infected recipient mice. (A) Intratracheally transplanted tdtomato^+^ EpiSPC (HA^+^ or HA^-^), applied into wt mice at d7 pi, were counted by microscopy. Random images were taken at d7 post transplantation. (B) Quantification of the red pixel area in PR/8-infected wt mice that were transplanted infected (HA^+^) or non-infected (HA^-^) tdtomato^+^ EpiSPC from infected donor tdtomato^+^ mice at d7 pi, or EpiSPC from non-infected tdtomato^+^ donor mice. Analyses was performed at d14 post transplantation. Bar graphs represent means ± SD of 30 randomly taken images/mouse; **p<0.01. (C) EpiSPC of non-infected tdtomato^+^ mice do not expand and generate tissue *de* novo when intratracheally transplanted into PR/8 infected wt mice at d7 pi. Images were taken at d14 post transplantation, bar = 100μm.
(TIF)
