# Supplementary figures and images for: Influenza Virus Infects Epithelial Stem/Progenitor Cells of the Distal Lung: Impact on Fgfr2b-Driven Epithelial Repair
Source: PLoS Pathog. 2016 Jun 20;12(6):e1005544. doi: 10.1371/journal.ppat.1005544 (PMC4913929; doi:10.1371/journal.ppat.1005544)

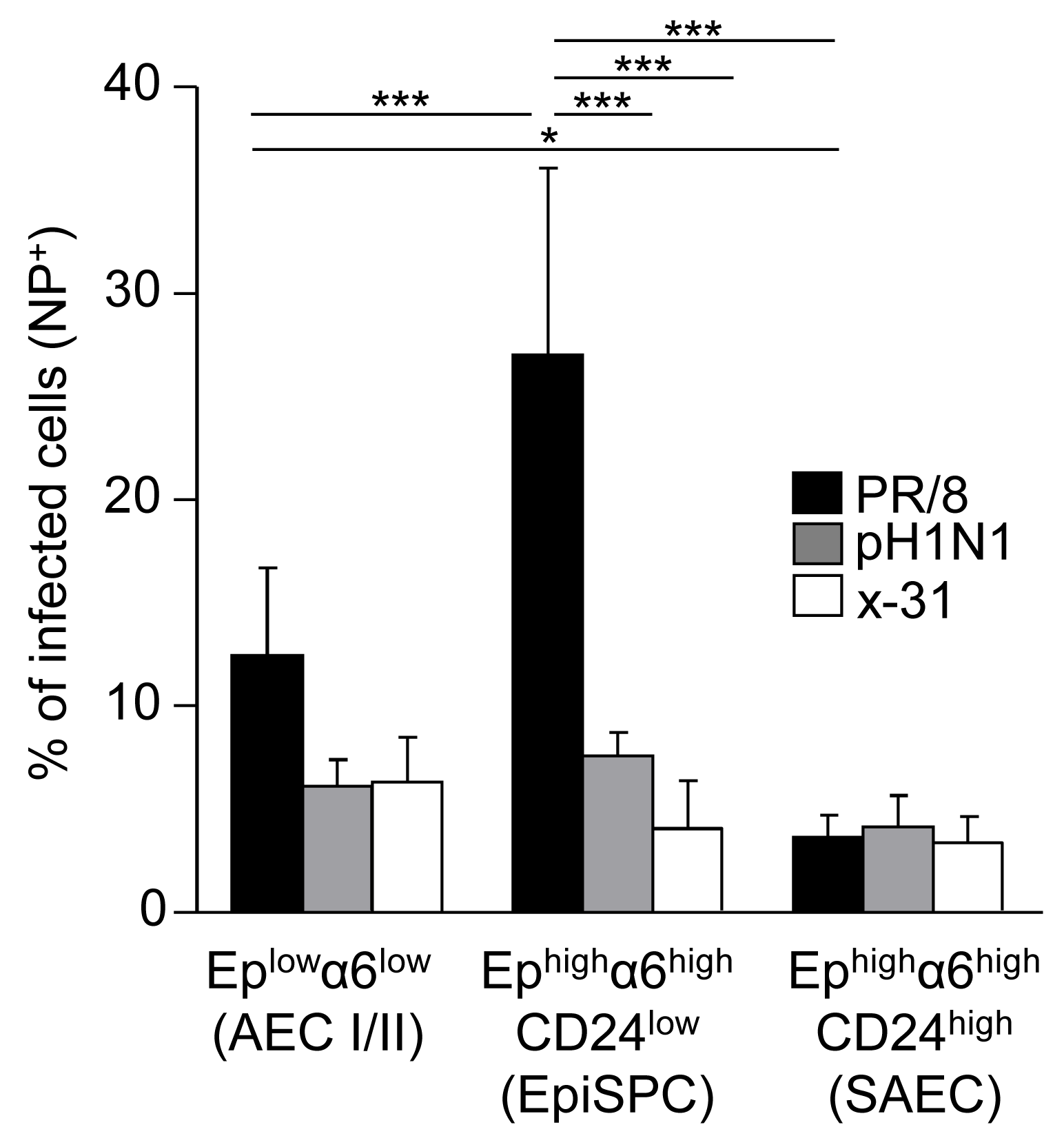

Supplement: S1 Fig — Flow sorted AEC, EpiSPC and SAEC were seeded in culture plates to equal densities and infected ex vivo with PR8, pH1N1 or H3N2 (x-31) at MOI 2, respectively. After 6h, infection rates were quantified by flow cytometric analysis by gating on nucleoprotein (NP)-positive fractions. Bar graphs show mean values ± SD for n = 3 individual experiments; * p<0.05; **p<0.01; ***p<0.001. (TIF) [file ppat.1005544.s001.tif]

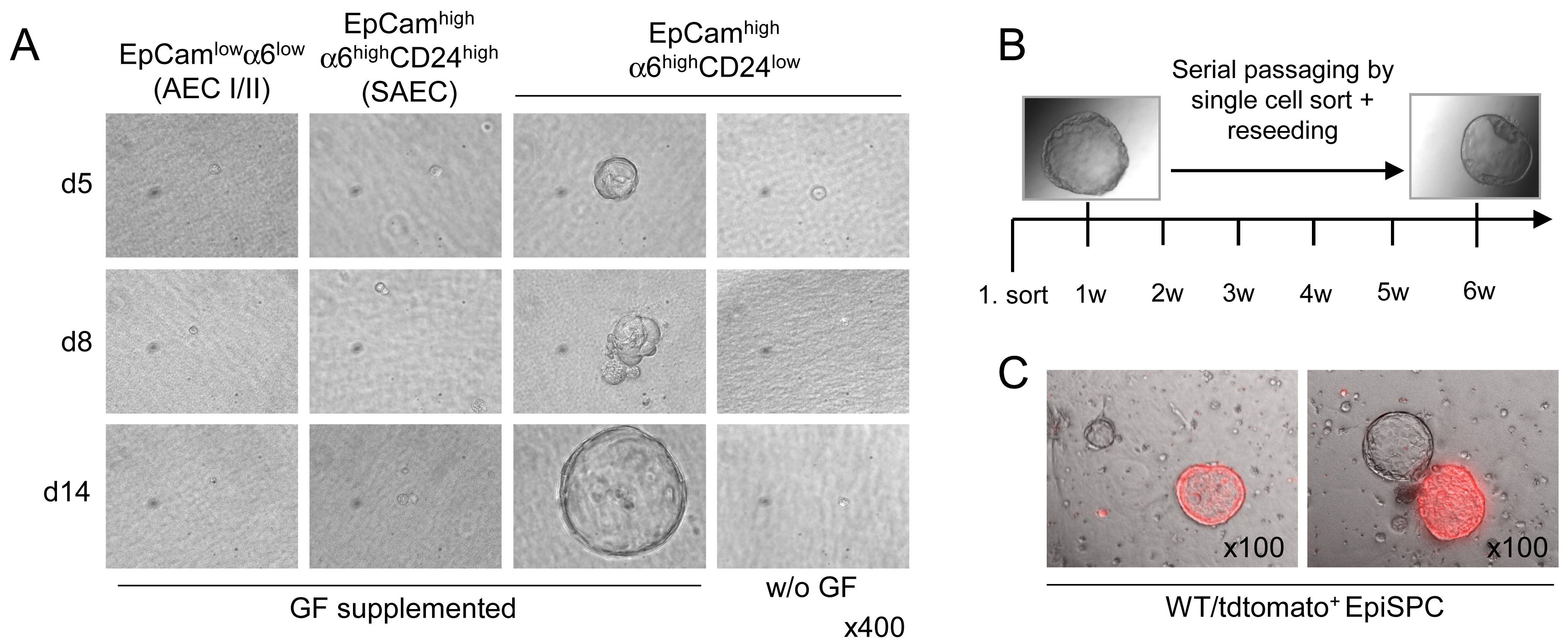

Supplement: S2 Fig — (A) AEC, SAEC and EpiSPC flow-sorted from wt mice were cultured in 3D matrix with or without (w/o) growth factor (GF) supplementation. (B) EpiSPC were single cell sorted into matrix using the BD ACDU (automated cell deposition unit), resulting in clonal expansion and organoid outgrowth. After one week of culture, cell suspensions were prepared from the organoids and single cells were reseeded. This was repeated for at least 6 times, demonstrating high clonal potential of EpiSPC. (C) Single cell suspensions of flow-sorted EpiSPC derived from tdtomato and wt mice were mixed and cultured in matrix, resulting in generation of either tdtomato+ or tdtomatoneg organoids. (TIF) [file ppat.1005544.s002.tif]

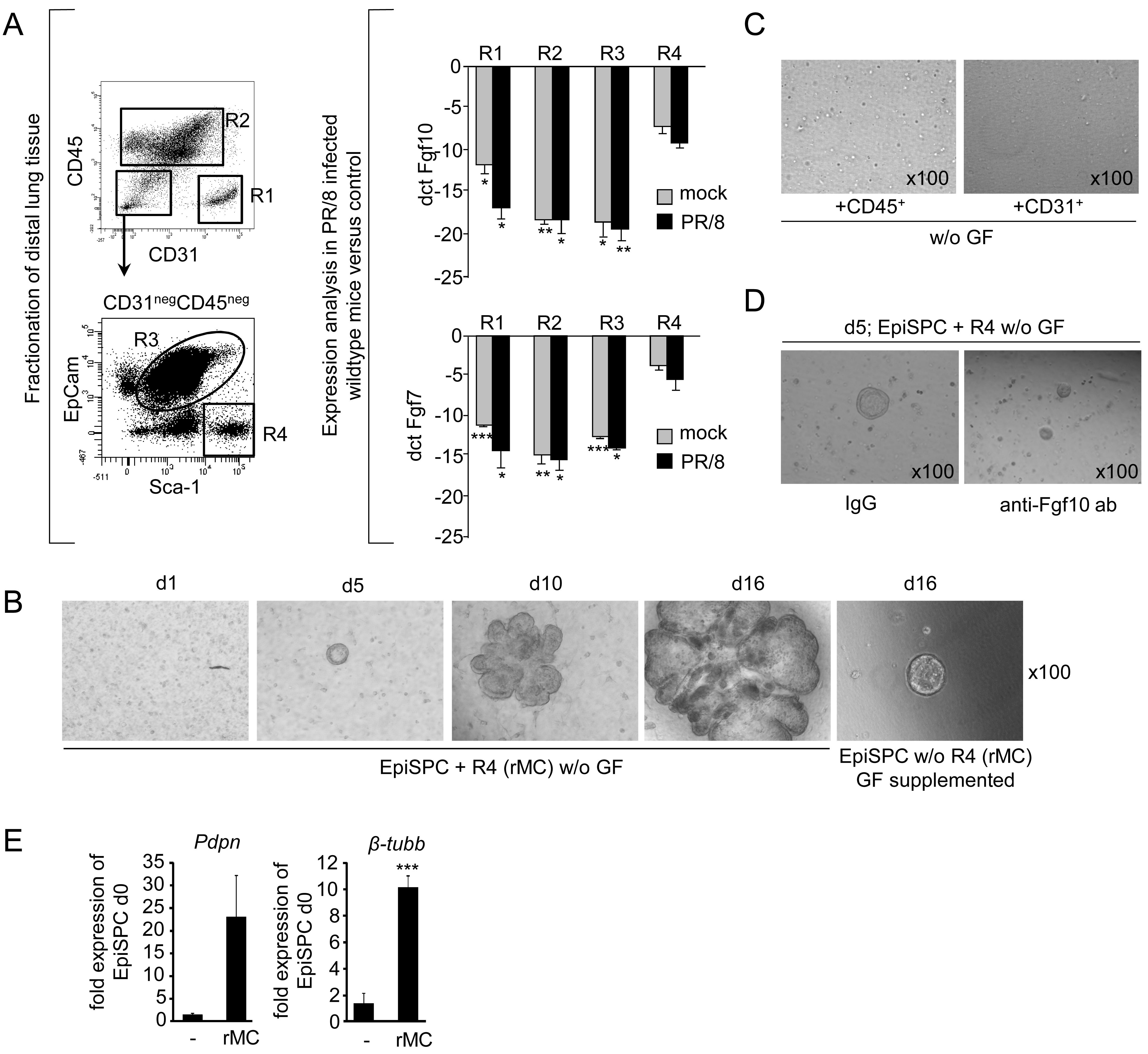

Supplement: S3 Fig — (A) Gating strategy of lung tissue for the separation of endothelial cells (R1), leukocytes (R2), epithelial cells (R3) and Sca-1high mesenchymal cells (R4). mRNA expression (ΔCT to housekeeping gene) of Fgf10 and Fgf7 in flow-sorted R1-R4 populations from mock or PR/8 infected wt mice. (B) Matrix co-culture of resident mesenchymal cells (rMC) and EpiSPC without growth factor supplementation results in generation of lung-like structures at d16 of culture. The left photomicrograph shows mono-cultured EpiSPC in the presence of growth factors. (C) Co-culture of EpiSPC with CD31+ endothelial (R1) cells or CD45+ leukocytes (R2) instead of rMC does not result in lung-like organoid formation at d10 of culture. (D) Organoid outgrowth in non-GF-supplemented rMC-EpiSPC co-cultures, is inhibited in presence of a neutralizing anti-Fgf10 ab, but not with corresponding isotype IgG ab, at d5 of culture. (E) After 10d of co-culture (rMC) versus GF-supplemented mono-culture (-), organoids were isolated and mRNA expression of marker genes of alveolar (T1α/podoplanin) and airway (β-tubulin) differentiation was quantified. Values are normalized to freshly flow-sorted EpiSPC, respectively. All bar graphs represent means ± SD of n = 3 independent experiments; * p<0.05; **p<0.01; ***p<0.001. Significances in (A) refer to the PR8 or mock group of R4, respectively. Photomicrographs are representative for n = 3 independent experiments. (TIF) [file ppat.1005544.s003.tif]

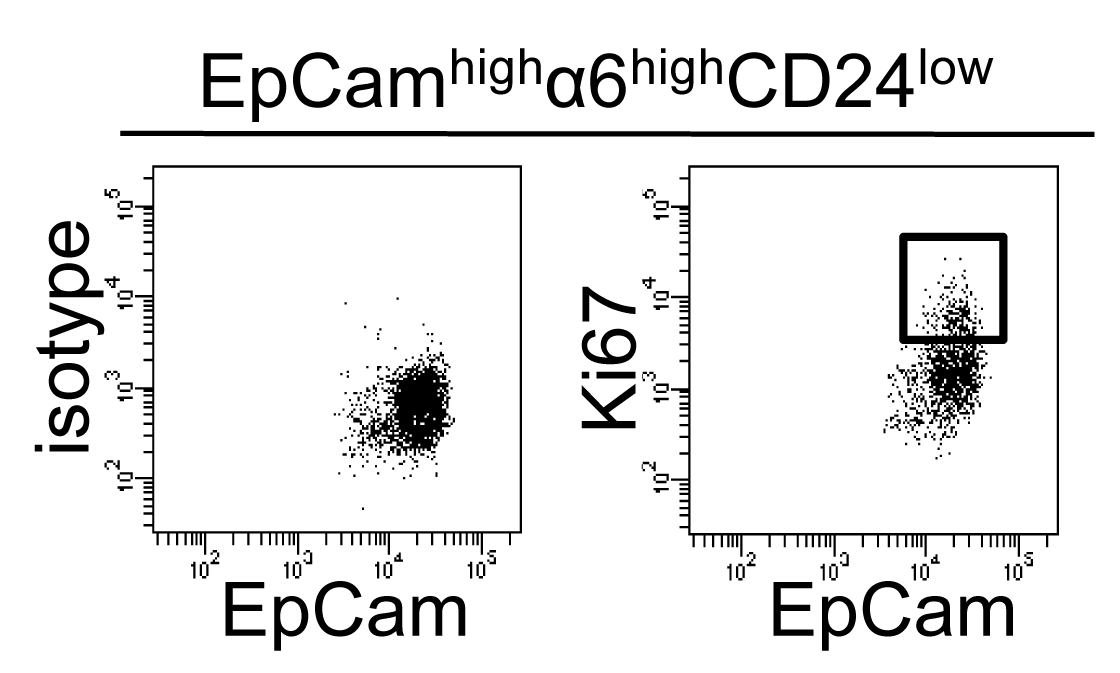

Supplement: S4 Fig — EpCamhighα6highCD24low cells were subgated for the Ki67+ fraction. Gates were set according to controls containing fluorochrome-labeled isotype control. (TIF) [file ppat.1005544.s004.tif]

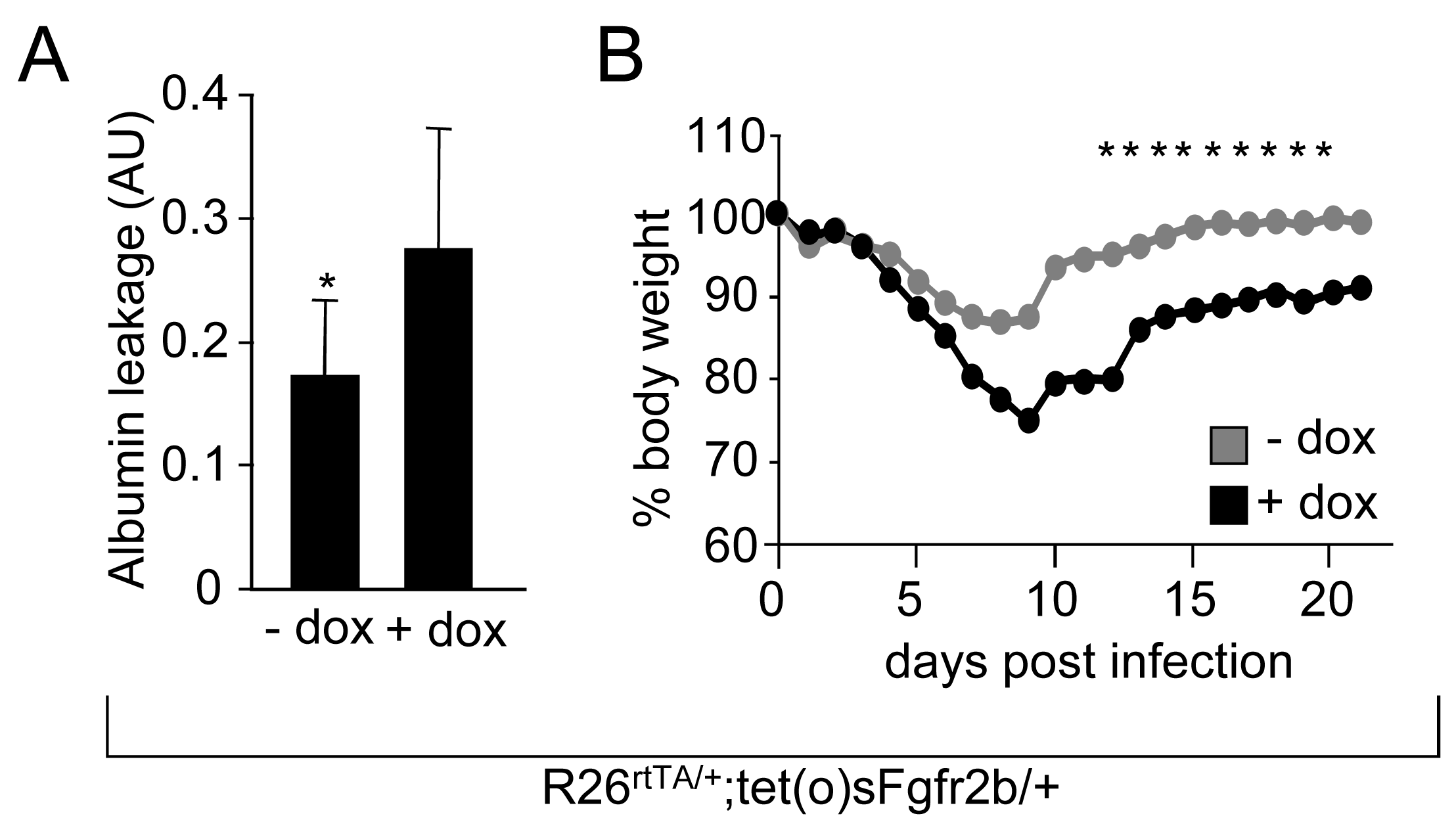

Supplement: S5 Fig — Doxycycline-induced or non-induced Rosa26 rtTA/+ ;tet(O)sFgfr2b/+ mice were infected with PR/8 and lung barrier function was analyzed by quantification of alveolar leakage of FITC-labeled albumin at d14 pi. Values are given in arbitrary units (AU) and represent ratios of FITC fluorescence in BALF and serum and are derived from n = 7 independent experiments (A). Body weight (B) of doxycycline-induced vs. non-induced Rosa26 rtTA/+ ;tet(O)sFgfr2b/+ mice (n = 8, respectively) after PR/8 infection were analyzed until d21 pi. Bar graphs represent means ± SD; * p<0.05; +dox, doxycycline food; -dox, normal diet. (TIF) [file ppat.1005544.s005.tif]

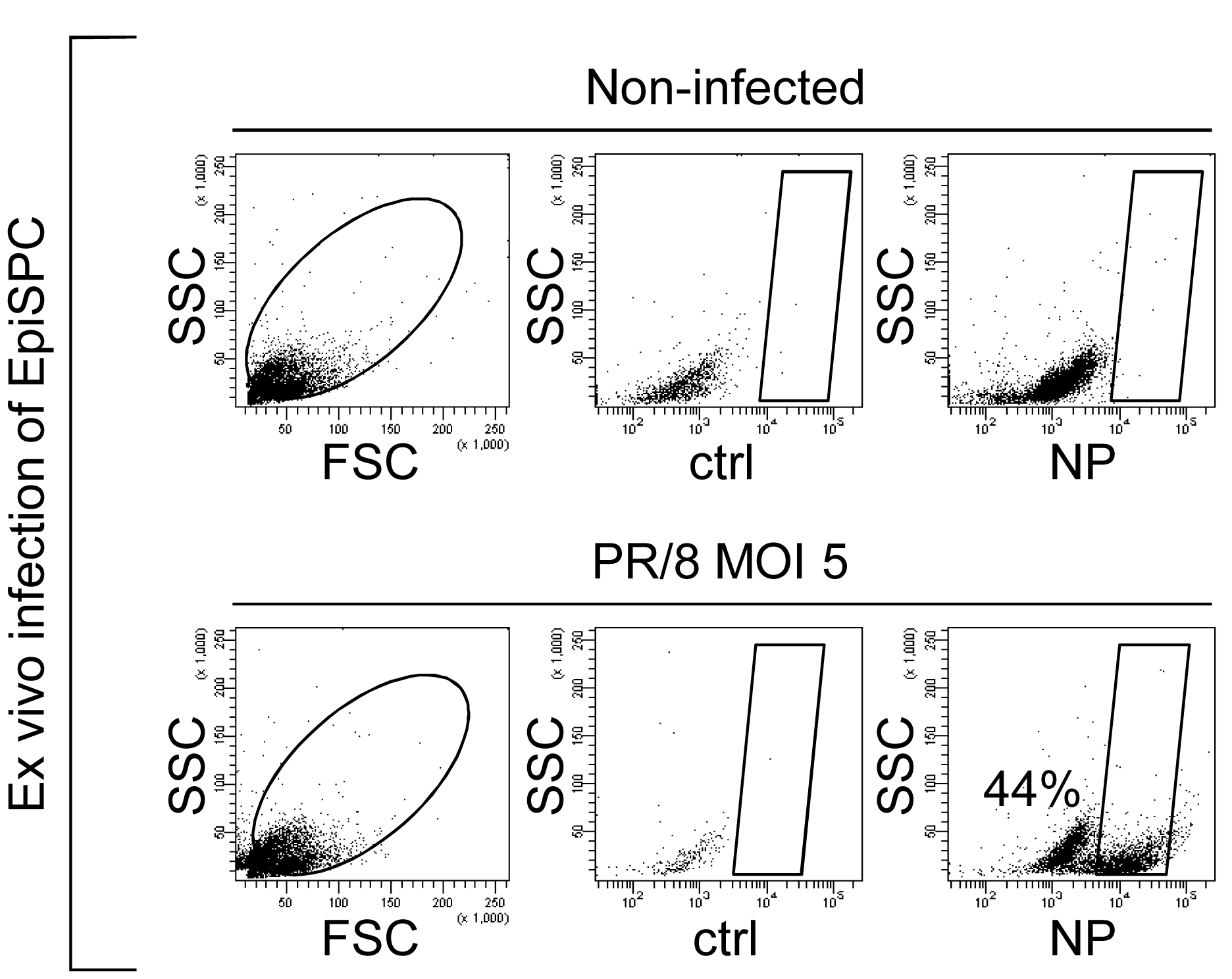

Supplement: S6 Fig — Flow-sorted EpiSPC in suspension were PR/8 infected for 8h at continuous rotation (bottom) or were left un-infected (top). The infected fractions were quantified by FACS using IV nucleoprotein (NP) staining (∼44% at MOI = 5). (TIF) [file ppat.1005544.s006.tif]

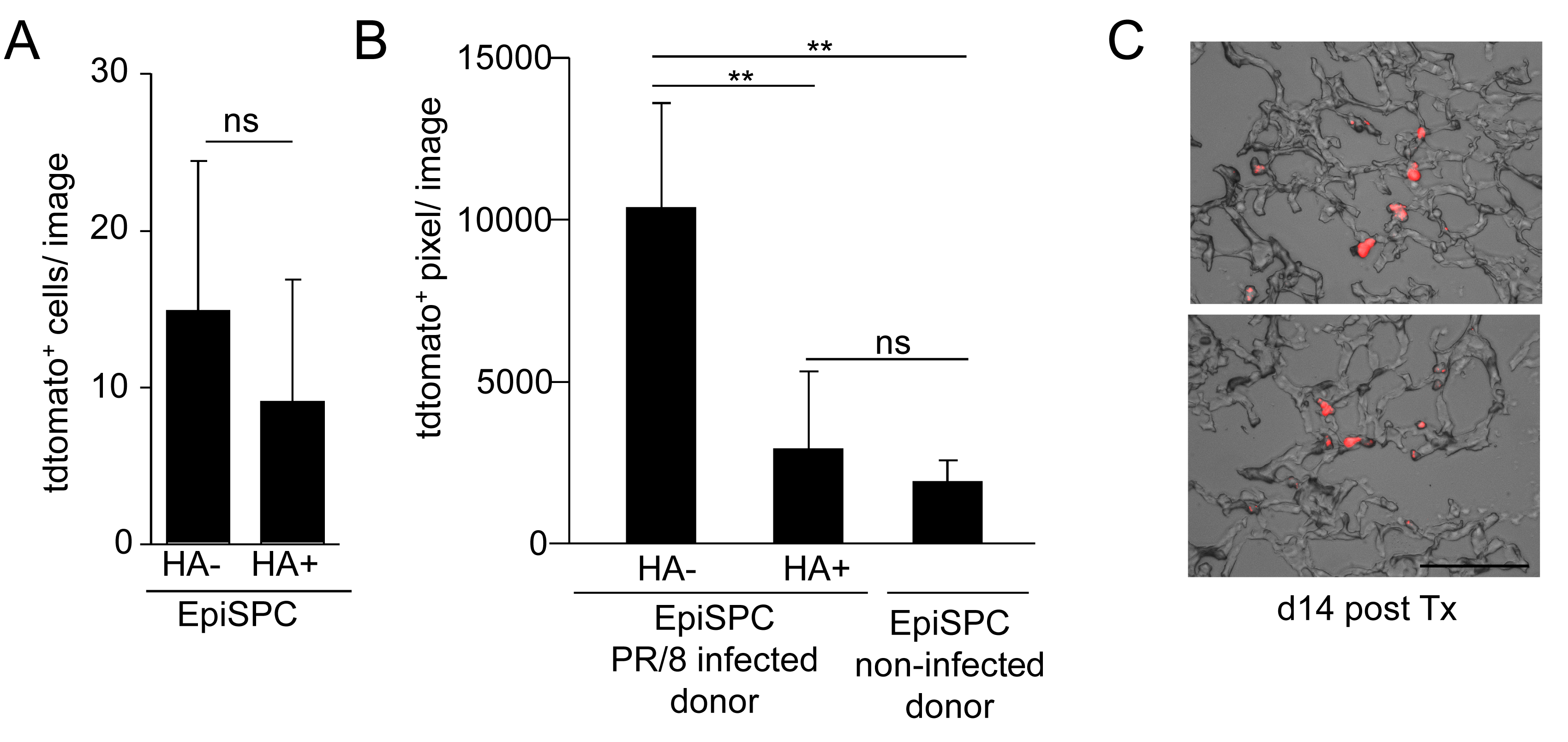

Supplement: S7 Fig — (A) Intratracheally transplanted tdtomato+ EpiSPC (HA+ or HA-), applied into wt mice at d7 pi, were counted by microscopy. Random images were taken at d7 post transplantation. (B) Quantification of the red pixel area in PR/8-infected wt mice that were transplanted infected (HA+) or non-infected (HA-) tdtomato+ EpiSPC from infected donor tdtomato+ mice at d7 pi, or EpiSPC from non-infected tdtomato+ donor mice. Analyses was performed at d14 post transplantation. Bar graphs represent means ± SD of 30 randomly taken images/mouse; **p<0.01. (C) EpiSPC of non-infected tdtomato+ mice do not expand and generate tissue de novo when intratracheally transplanted into PR/8 infected wt mice at d7 pi. Images were taken at d14 post transplantation, bar = 100μm. (TIF) [file ppat.1005544.s007.tif]
